# TGF-β1 protein trap AVID200 beneficially affects hematopoiesis and bone marrow fibrosis in myelofibrosis

**DOI:** 10.1172/jci.insight.145651

**Published:** 2021-09-22

**Authors:** Lilian Varricchio, Camelia Iancu-Rubin, Bhaskar Upadhyaya, Maria Zingariello, Fabrizio Martelli, Paola Verachi, Cara Clementelli, Jean-Francois Denis, Adeeb H. Rahman, Gilles Tremblay, John Mascarenhas, Ruben A. Mesa, Maureen O’Connor-McCourt, Anna Rita Migliaccio, Ronald Hoffman

**Affiliations:** 1 Tisch Cancer Institute,; 2 Department of Pathology, Molecular and Cell-Based Medicine, and; 3 Human Immune Monitoring Core, Icahn School of Medicine at Mount Sinai, New York, New York, USA.; 4 Unit of Microscopic and Ultrastructural Anatomy, Department of Medicine, University Campus Bio-Medico, Rome, Italy.; 5 National Center for Drug Research and Evaluation, Istituto Superiore di Sanità, Rome, Italy.; 6 Biomedical and Neuromotorial Sciences, Alma Mater University, Bologna, Italy.; 7 Forbius, Saint-Laurent, Montreal, Quebec, Canada.; 8 Mays Cancer Center, San Antonio, Texas, USA.

**Keywords:** Hematology, Hematopoietic stem cells, Leukemias

## Abstract

Myelofibrosis (MF) is a progressive chronic myeloproliferative neoplasm characterized by hyperactivation of JAK/STAT signaling and dysregulation of the transcription factor GATA1 in megakaryocytes (MKs). TGF-**β** plays a pivotal role in the pathobiology of MF by promoting BM fibrosis and collagen deposition and by enhancing the dormancy of normal hematopoietic stem cells (HSCs). In this study, we show that MF-MKs elaborated significantly greater levels of TGF-**β**1 than TGF-**β**2 and TGF-**β**3 to a varying degree, and we evaluated the ability of AVID200, a potent TGF-**β**1/TGF-**β**3 protein trap, to block the excessive TGF-**β** signaling. Treatment of human mesenchymal stromal cells with AVID200 significantly reduced their proliferation, decreased phosphorylation of SMAD2, and interfered with the ability of TGF-**β**1 to induce collagen expression. Moreover, treatment of MF mononuclear cells with AVID200 led to increased numbers of progenitor cells (PCs) with WT *JAK2* rather than mutated *JAK2V617F*. This effect of AVID200 on MF PCs was attributed to its ability to block TGF-**β**1–induced p57^Kip2^ expression and SMAD2 activation, thereby allowing normal rather than MF PCs to preferentially proliferate and form hematopoietic colonies. To assess the in vivo effects of AVID200, *Gata1^lo^* mice, a murine model of MF, were treated with AVID200, resulting in the reduction in BM fibrosis and an increase in BM cellularity. AVID200 treatment also increased the frequency and numbers of murine progenitor cells as well as short-term and long-term HSCs. Collectively, these data provide the rationale for TGF-**β**1 blockade, with AVID200 as a therapeutic strategy for patients with MF.

## Graphical abstract

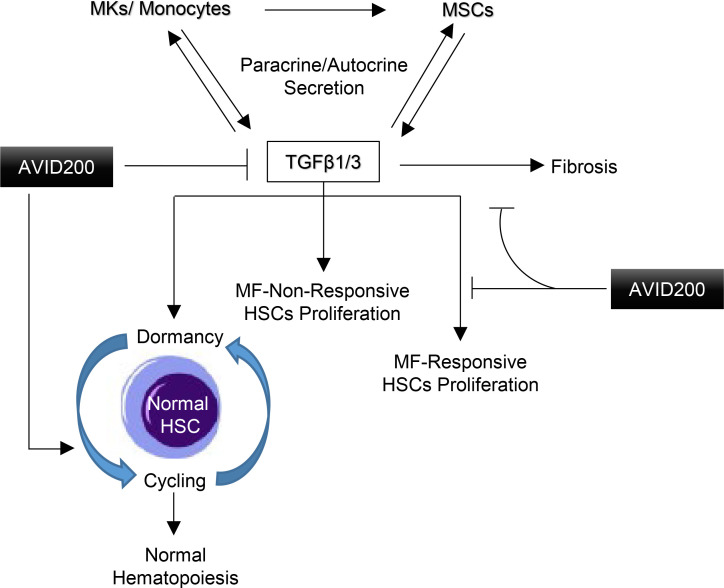

## Introduction

Myelofibrosis (MF) is a clonal hematopoietic stem cell (HSC) disorder for which there are limited therapeutic options. Patients with symptomatic disease have improvement in their quality of life with the administration of JAK1/2 inhibitors (1–3). Although these medications are frequently effective, they have a limited impact on prolonging survival, and eventually patients frequently become intolerant or refractory to their action (4). Presently, the only curative approach for patients with MF remains allogeneic HSC transplantation (5). These limitations have led to a search for alternative drugs that might affect other relevant MF pathological pathways.

MF is characterized by BM megakaryocyte (MK) hyperplasia. These MKs release excess proinflammatory cytokines and growth factors, altering the hematopoietic microenvironment in a manner that supports MF HSC/hematopoietic progenitor cell (HSC/HPC) predominance (6–8). Among the proinflammatory cytokines, TGF-β plays a dual role in promoting BM fibrosis and disturbing the balance between normal and malignant hematopoiesis, which are morphologic hallmarks of this myeloproliferative neoplasm (MPN) (9). MKs from patients with MF generate greater amounts of TGF-β than those from other MPNs (6). However, MKs are not the only source of TGF-β in MF because macrophages and monocytes as well as other accessory cell types elaborate increased levels of this cytokine (10). There are 3 isoforms of TGF-β (TGF-β1; ref. 11; TGF-β2; ref. 12; and TGF-β3; ref. 13) that share 70%–80% of their amino acid sequence identity, but have distinct biological properties. Active forms of TGF-β1 bind to a membrane receptor serine/threonine kinase complex that phosphorylates receptor SMADs (SMAD2 and SMAD3), which accumulate in the nucleus where they complex with SMAD4 to regulate target gene expression (14). TGF-β can also signal through noncanonical pathways, including ERK, p38 MAPK and JNK activation (15, 16). TGF-β1 and TGF-β3 have additional effects, including their ability to inhibit normal hematopoiesis through the canonical SMAD-dependent signaling pathway, which induces normal HSC quiescence (17–19). Neutralization of TGF-β1 has been shown to release early progenitor cells from their quiescent status (20). TGF-β1 partially prevents HSC reentry into the cell cycle and hematopoietic regeneration by upregulating transcription of the cyclin-dependent kinase inhibitor p57^Kip2^ (19, 21) and pSMAD2 (22).

Various mouse models have been developed and used to establish the role of TGF-β in the pathogenesis of fibrosis (23–29). Mice carrying a hypomorphic mutation in the transcription factor GATA1 (*Gata1^lo^*) develop MF over time and have been used by several investigators as a mouse model of human MF (28). Hyperactive thrombopoietin (TPO) signaling has been reported to dysregulate *GATA1* (30), leading to human MF-MKs with reduced GATA1 levels (28).

Because sustained secretion of TGF-β has been proposed to play a central role in the pathogenesis and progression of several human disorders associated with fibrosis, a number of strategies have been evaluated targeting aberrant TGF-β signaling. MF is an ideal neoplasm to evaluate such therapeutic agents because it is characterized by sustained secretion of TGF-β, which contributes not only to BM fibrosis but also to the depletion of the pool of normal HSCs. Gastinne et al., using the TPO overexpressing mouse model of MF, have previously shown that the administration of a TGF-β soluble receptor sequestered TGF-β1 and reversed BM and splenic fibrosis (31).

In this report, we evaluate the potential therapeutic efficacy of targeting TGF-β in MF with AVID200, a potent TGF-β trap with antibody-like properties and picomolar (pM) potency against TGF-β1 and TGF-β3 and minimal activity against TGF-β2. This specificity is desirable because TGF-β2 is a known positive regulator of hematopoiesis (32) and is closely associated with maintenance of normal cardiovascular function (33).

## Results

### AVID200 is a TGF-β trap with antibody-like properties.

The specificity of AVID200 was demonstrated using A549 lung adenocarcinoma cells, which release IL-11 in response to recombinant (r) TGF-β (rTGF-β; ref. 34). AVID200 neutralized rTGF-β1 and rTGF-β3 activity with similar, low pM, IC_50_ values of 1.4 pM and 4.1 pM, respectively. In contrast, the ability of AVID200 to neutralize the effect of rTGF-β2 was almost 4 orders of magnitude lower (IC_50_ of 4900 pM; Supplemental Figure 1; supplemental material available online with this article; https://doi.org/10.1172/jci.insight.145651DS1).

### Effects of AVID200 on TGF-β1 in MKs derived by normal donors and MF mononuclear cells.

**Figure 1:**
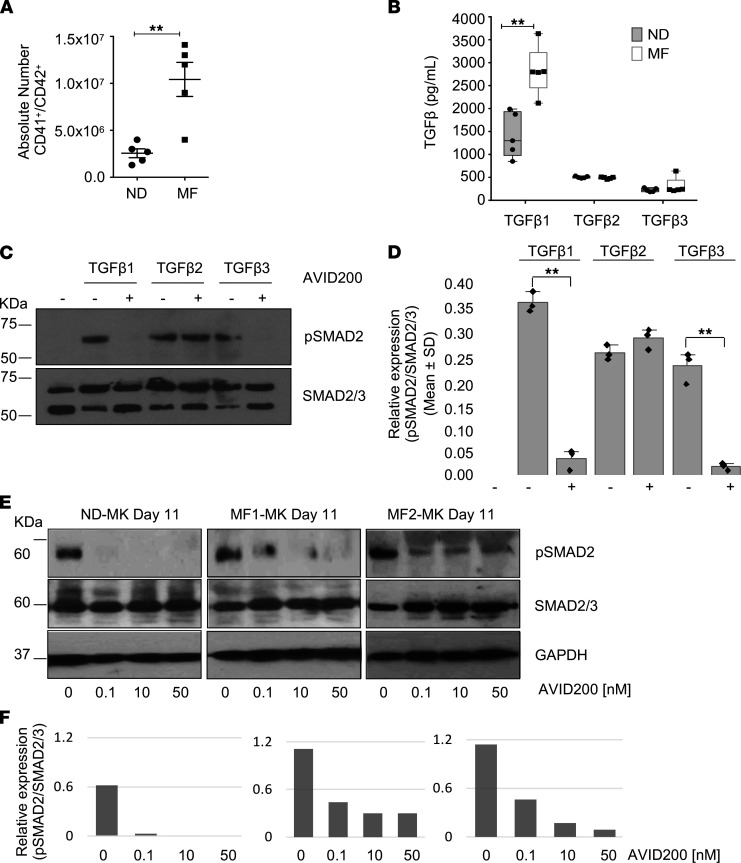
AVID200 blocks the effect of exogenous TGF-β1/TGF-β3 as well TGF-β produced in an autocrine fashion. (**A**) Absolute number of MKs (CD41/CD42) generated in cultures of MF and ND MNCs ± SEM (*n* = 5). The absolute number of MF-MKs was significantly greater than ND-MKs (**P < 0.01). (**B**) The levels of TGF-β1, TGF-β2, and TGF-β3 were quantified in media conditioned by ND and MF MNCs under MK differentiation conditions (day 11). Results are displayed as pg/mL of culture supernatant ± SEM (*n* = 5). Significantly greater levels of TGF-β1 were observed in the MF-MK CM (**P *<* 0.01, by ANOVA). (**C**) MNCs were cultured for 48 hours in the presence of recombinant TGF-β1, TGF-β2, or TGF-β3 (10 ng/mL) with and without AVID200 (50 nM). Western blot for pSMAD2 and total SMAD2/SMAD3 of the MNC cell lysate (*n* = 3). (**D**) Relative expression of pSMAD2 toward the corresponding SMAD2/SMAD3 band by Image Studio Lite 5.2 (**P *<* 0.01, by ANOVA). (**E**) Western blot for pSMAD2 and total SMAD2/SMAD3. GAPDH was used as an internal loading control for ND-MK and MF-MK cells. MK cultures were exposed on day 9 of differentiation to increasing concentrations of AVID200 for 48 hours. (**F**) Relative expression of pSMAD2 toward the corresponding SMAD2/SMAD3 band previously normalized toward GAPDH by Image Studio Lite 5.2. MF, myelofibrosis; MK, megakaryocyte; ND, normal donor; MNC, mononuclear cell; CM, conditioned media.

Because MKs are major sources of TGF-β in MF (6, 35), we generated cell populations enriched in MKs by culturing PMNCs obtained from normal donors (NDs) and patients with MF for 11 days as previously described (36). The frequency of MKs was determined by anti-CD41 and anti-CD42 antibody staining (Supplemental Figure 2A). The corresponding morphology of these MKs was evaluated on days 7, 9, and 11 of culture, which spans the transition from megakaryoblasts to mature MKs (Supplemental Figure 2B). Although the percentage of cells expressing CD41 and CD42 from ND and MF mononuclear cells (MNCs) were similar after 11 days, the absolute number of MF CD41^+^/CD42^+^ MKs was significantly greater than ND-MKs (*P* = 0.003; Figure 1A). Significantly greater levels of TGF-β1 were observed in the MF-MK conditioned media (CM) than the ND-MK CM (*P* = 0.0026). Moreover, the levels of TGF-β2 and TGF-β3 were lower than TGF-β1 in both ND-MK and MF-MK CM (Figure 1B). We measured the effects of rTGF-β1, rTGF-β2, and rTGF-β3 (in the presence and absence of AVID200) on the phosphorylation of SMAD2 (pSMAD2 Ser465/467) in MF MNCs after 4 days of culture (Supplemental Figure 3). AVID200 specifically blocked the activation of SMAD2 by rTGF-β1 and rTGF-β3 but not rTGF-β2, confirming the specificity of AVID200 (Figure 1, C and D).

We measured pSMAD2 to explore the endogenous activation of TGF-β signaling in ND or MF cells enriched for MKs. The effect of increasing concentrations of AVID200 was previously evaluated on MF-MK by assessing pSMAD2 status as readout for TGF-β pathway activation. AVID200 (10 nM) decreased the levels of pSMAD2 by 2-fold (Supplemental Figure 4). A further increase in the concentration of AVID200 by 5-fold (50 nM), which was not associated with any toxicity, completely abolished the activation of SMAD2. Treatment, however, with a greater concentration of AVID200 did not result in a further decrease in pSMAD2, but affected the activity of the positive regulator of hematopoiesis TGF-β2, as shown in Supplemental Figure 1. Therefore, to rule out any off-target effects of AVID200, we used 50 nM as the highest dose.

Robust expression of pSMAD2 was observed in the absence of an exogenous source of TGF-β (Figure 1, E and F). Addition of AVID200 to ND-MK and MF-MK cultures decreased pSMAD2 levels without affecting total SMAD2/SMAD3 levels, indicating that AVID200 blocks the effects of TGF-β produced in a paracrine/autocrine fashion (Figure 1, E and F).

### AVID200 antagonized TGF-β1–induced proliferation and collagen production by human mesenchymal stromal cells.

**Figure 2:**
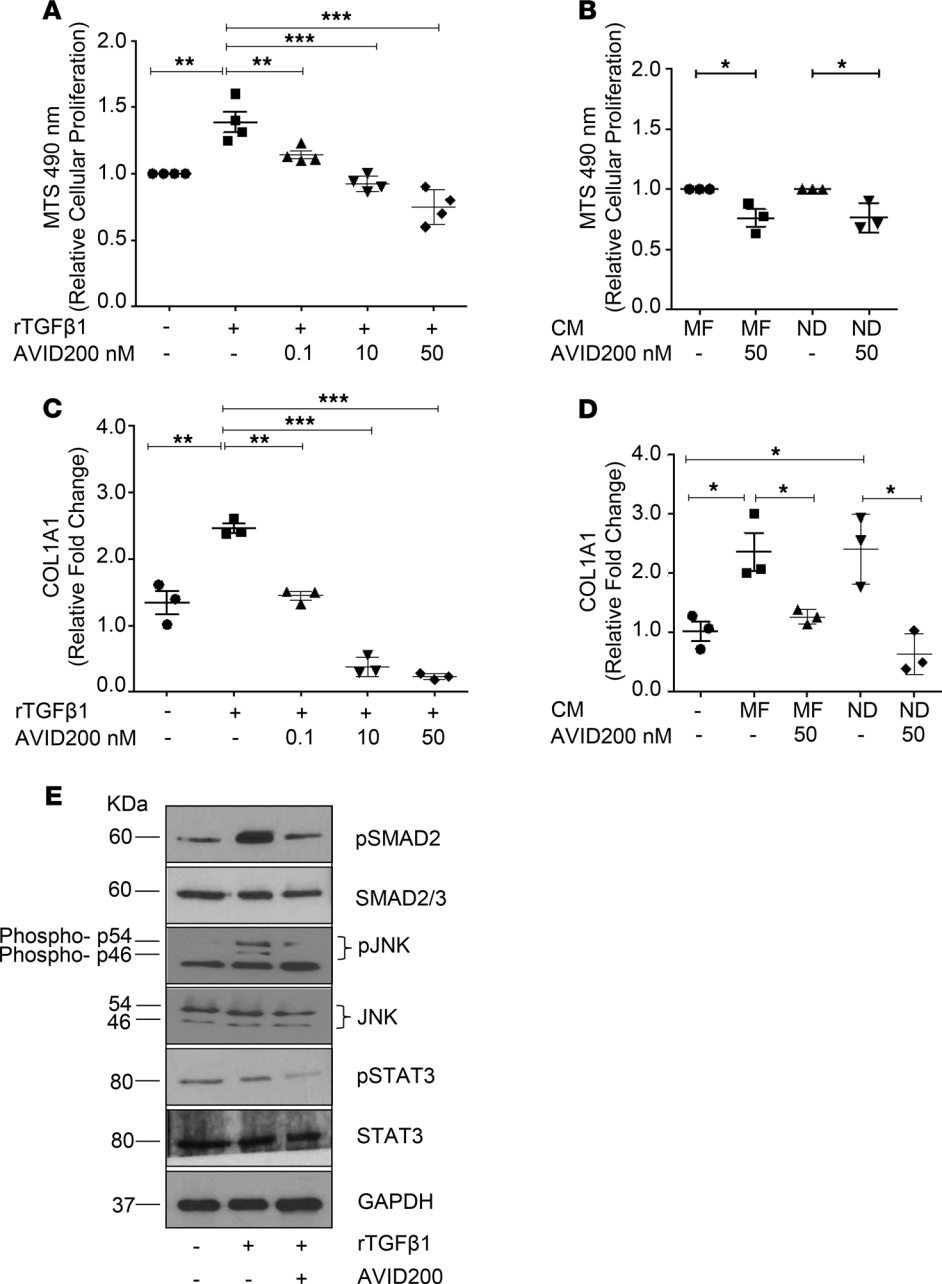
Cellular proliferation and collagen gene expression by normal human MSCs in the presence of recombinant TGF-β1 or MK CM. (**A** and** B**) Proliferation of ND BM MSCs treated with 1 ng/mL of rTGF-β1, or CM from MF-MK/ND-MK, alone, or in combination with varying concentrations of AVID200. After 48 hours, absorbance was measured using a plate reader at 490 nm (*P < 0.05, **P < 0.01, ***P < 0.001, by ANOVA, multiple comparison using TGF-β1 or CM as control). (**C** and** D**) BM MSCs were cultured in the absence or presence of 1 ng/mL of rTGF-β1 or in the presence of CM from MF-MK/ND-MK cultures alone or in combination with varying concentrations of AVID200 for 72 hours. Human collagen I (*COL1A1*) mRNA levels were measured by qRT-PCR (*P < 0.05, **P < 0.01, ***P < 0.001, ****P < 0.0001, by ANOVA). (**E**) Western blot of the same cells in **C** treated with 1 ng/mL rTGF-β1 with and without 50 nM AVID200 for pSMAD2, SMAD2/SMAD3, pJNK, JNK, pSTAT3, and STAT3 expression. GAPDH was loaded as an internal control (*n* = 3 biological replicates). MF, myelofibrosis; MK, megakaryocyte; ND, normal donor; MNC, mononuclear cell; CM, conditioned media; MSC, mesenchymal stromal cells.

Due to its pleiotropic role, TGF-β has different effects that are cell type–specific (37–39). TGF-β1 is known to induce the proliferation of human BM mesenchymal stromal cells (MSCs) and the deposition of collagen with a consequent fibroblast proliferation and matrix production (37, 38, 40). Cellular proliferation of normal MSCs was evaluated after treatment with human rTGF-β1, or CM from ND-MK and MF-MK cultures, in the presence or absence of AVID200. rTGF-β1 increased the proliferation of normal MSCs (*P* < 0.01), which returned to basal levels with the addition of increasing concentrations of AVID200 (*P* < 0.001; Figure 2A). AVID200 had similar effects on MSCs cultured with CM from ND-MK or MF-MK cultures (*P* < 0.05; Figure 2B). These data indicate that AVID200 directly blocks the effect of rTGF-β1, as well as the TGF-β1 present in MK-CM. MF stroma is also characterized by an increase in type I collagen (*COL1A1*; ref. 35) deposition. Because normal (WT) MSCs respond to TGF-β1 by also increasing collagen synthesis mimicking the fibrotic features of MSCs derived from MF (41), we used normal MSCs to test the effect of AVID200 on collagen production. The treatment of normal MSCs with rTGF-β1 increased *COL1A1* expression (*P* < 0.01) and the addition of AVID200 eliminated the TGF-β–mediated increase in *COL1A1* expression in a dose-dependent manner (*P* < 0.001; Figure 2C). MF-MK and ND-MK CM also increased *COL1A1* expression by MSCs (*P* < 0.05), which was eliminated by the addition of AVID200 treatment (*P* < 0.05), indicating that TGF-β1 within the CM was responsible for these effects (Figure 2D). We next demonstrated that rTGF-β1 activated phosphorylation of SMAD2 in MSCs without affecting total SMAD2/SMAD3 expression and that rTGF-β1–mediated SMAD2 phosphorylation was reduced by AVID200 (Figure 2E). The protein JNK pathway has also been reported to contribute to the development of fibrosis in several cellular systems (42, 43). JNK (p46 and p54) was phosphorylated (Thr183 and Tyr185) in response to the addition of rTGF-β1 and the degree of phosphorylation was reduced by AVID200 (Figure 2E). These findings indicate that AVID200 suppresses both canonical and noncanonical TGF-β pathways in normal MSCs. JAK2/STAT3 activation has been previously associated with the ability of TGF-β to induce pulmonary and liver fibrosis, epithelial-mesenchymal transition, and cancer cell migration/invasion (44–47). AVID200 treatment also decreased pSTAT3 levels in rTGF-β1–treated MSCs (Figure 2E). Taken together, our findings indicate that AVID200 could have affected multiple signaling pathways in MSCs and that crosstalk between these pathways likely led to fibrosis and collagen production, which can be blocked by AVID200.

### AVID200 treatment restored rTGF-β1–mediated suppression of normal hematopoiesis by inhibiting the pSMAD2 network.

**Figure 3:**
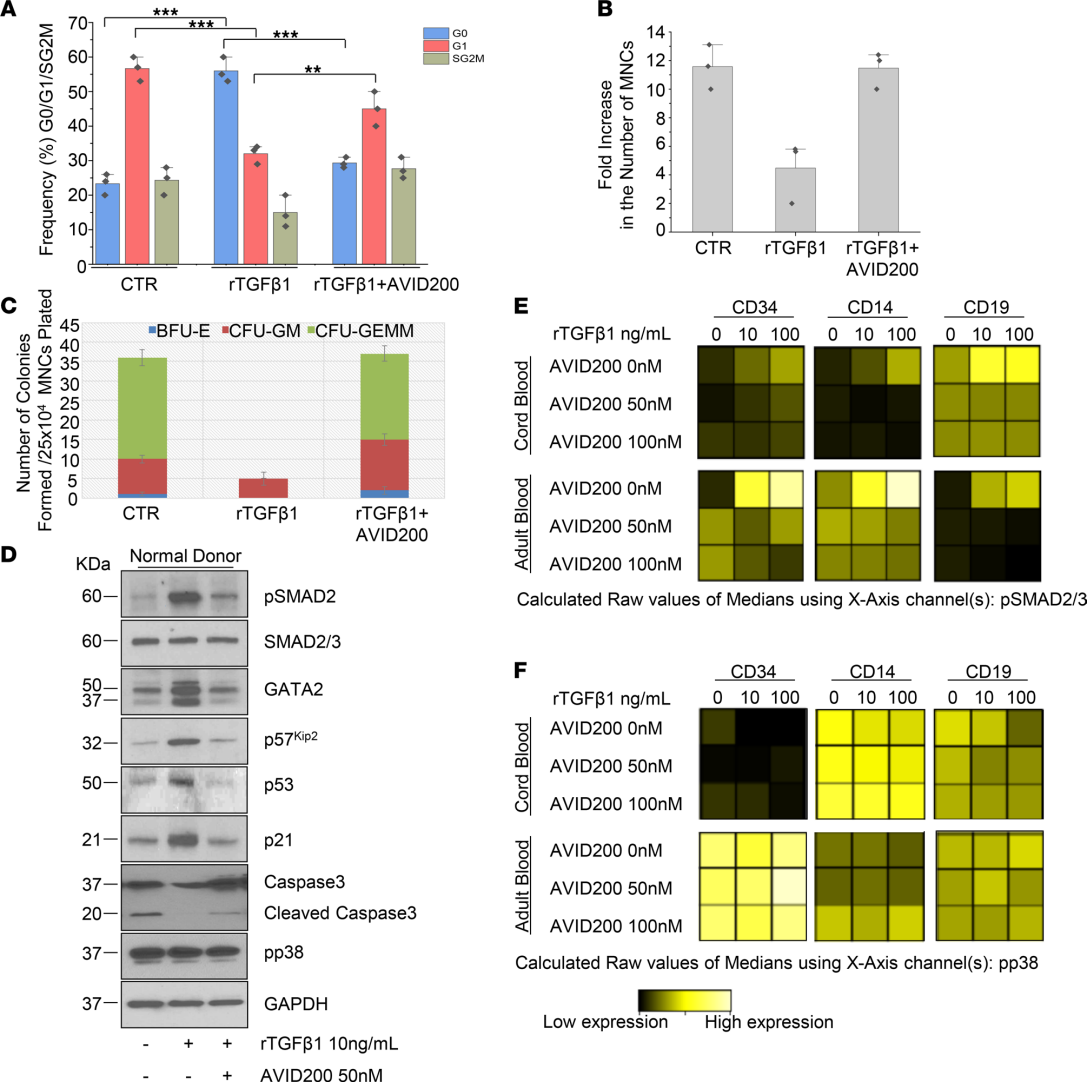
AVID200 reverses the G0-G1 blockade caused by rTGF-β1 and increases the clonogenicity of ND MNC cells. (**A**) The bar graph shows the percentages SD of ND MNCs in G0, G1, SG2M after treatment with 10 ng/mL rTGF-β1 alone or in combination with 50 nM AVID200 for 48 hours. The cells were stained with Hoechst 33342 and Pyronin Y. *n* = 3 different donors (** *P* < 0.01, *** *P* < 0.001, by ANOVA). (**B**) Fold increase of ND MNCs cultured for 6 days with cytokines alone (CTR) or in the presence of 10 ng/mL rTGF-β1 with or without 50 nM AVID200. Results are presented as the fold increase with respect to day 0 and are presented as mean ± SD of results from experiments performed with 3 different donors. (**C**) Number of colonies assayed in duplicate from cultures 25 × 10^4^ ND MNCs containing a cytokine combination (CTR) and 10 ng/ml rTGF-β1 alone or in combination with 50 nM AVID200. Results are presented as mean ± SD obtained in 2 separate experiments, using 2 different donors. (**D**) Molecular networks targeted by TGF-β in MNCs obtained from NDs. Representative WB analyses of pSMAD2, SMAD2/SMAD3, GATA2, p57^Kip2^, p53, p21, caspase 3, and pp38 of total extracts derived from ND treated with rTGF-β1 and AVID200 alone or in combination for 48 hours as indicated. GAPDH was used as loading control (*n* = 3 different donors). (**E**) CyTOF viSNE analysis for downstream effectors of TGF-β signaling, pSMAD2/pSMAD3, and (**F**) pp38 levels in CD34^+^, CD14^+^, and CD19^+^ gated cell populations from healthy donors (cord and adult blood; *n* = 2 different donors) previously treated with 10 or 100 ng/mL rTGF-β1 alone or in combination with 50 or 100 nM AVID200 for 30 minutes. ND, normal donor; MNC, mononuclear cell; viSNE, visualization of stochastic neighbor embedding.

**Figure 4:**
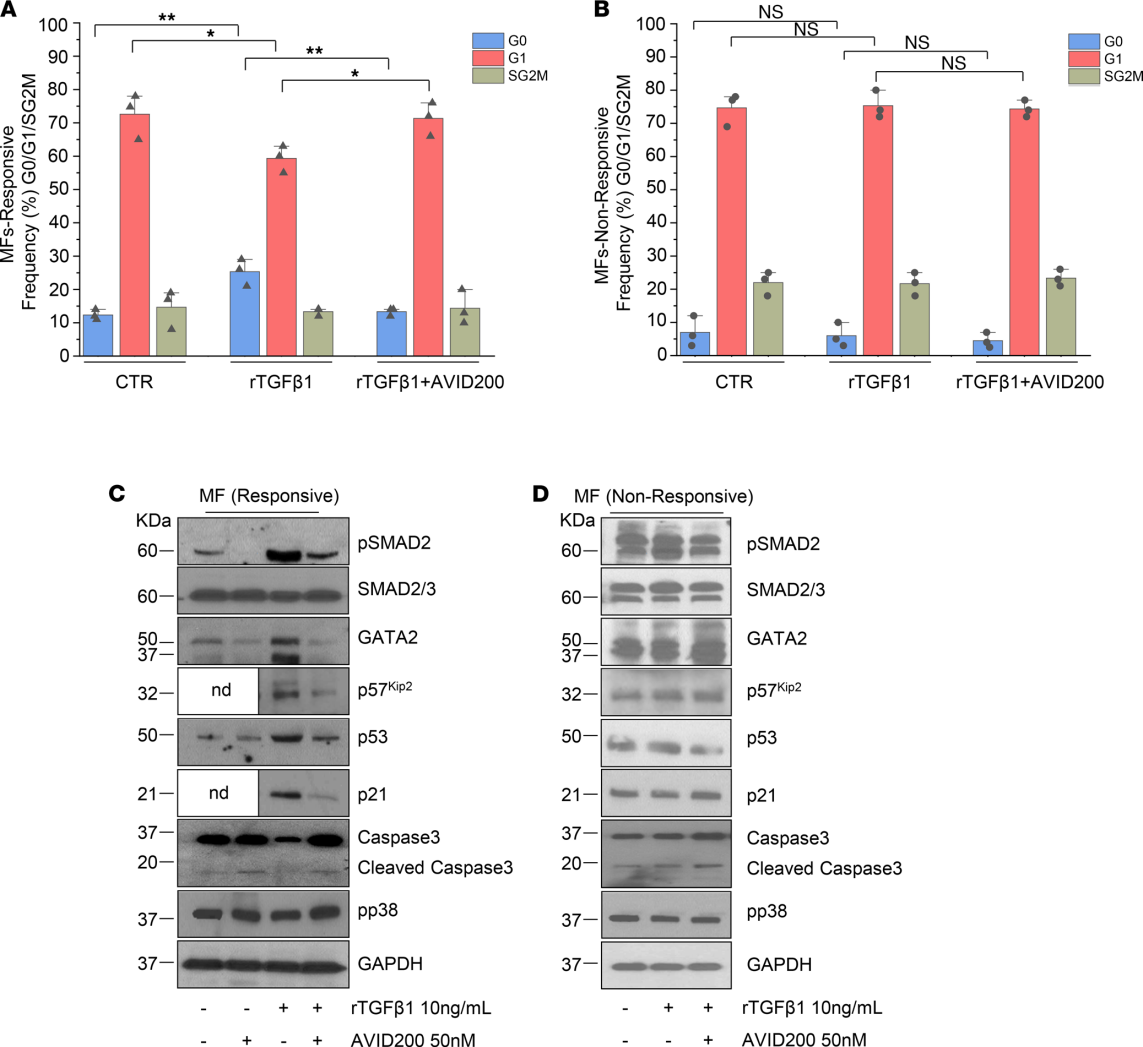
Molecular networks targeted by TGF-β in MNCs obtained from patients with MF. (**A** and** B**) The bar graph shows the percentages SD of MF MNCs in G0, G1, SG2M after treatment with 10 ng/mL rTGF-β1 alone or in combination with 50 nM AVID200 for 48 hours. The cells were stained with Hoechst 33342 and Pyronin Y (6 total different donors). The patients were felt to be responsive or unresponsive to rTGF-β1 based on the cycling status of MF MNCs after exposure to rTGF-β1 (*P < 0.05, **P < 0.01, NS, by ANOVA). (**C** and** D**) Representative WB analyses of pSMAD2, SMAD2/SMAD3, GATA2, p57^Kip2^, p53, p21, caspase 3, and pp38 of total extracts derived from MF MNCs treated with rTGF-β1 and AVID200 alone or in combination for 48 hours as indicated. GAPDH was used as loading control. Representative of 4 and 2 different donors in **C** and **D, **respectively. nd, not done; MF, myelofibrosis; MNC, mononuclear cell.

To determine if TGF-β1 influences normal HSC/HPC quiescence, we examined the cell cycle status of ND MNCs. rTGF-β1 increased the fraction of ND MNC cells in G0 from 23% ± 3% to 56% ± 3.5% (*P* < 0.001) and concomitantly decreased the G1 fraction by 1.8-fold (from 56.5% ± 3.5% to 31% ± 2.5%; *P* < 0.001) in comparison to untreated cells (CTR). These effects were eliminated by the addition of AVID200 (*P* < 0.01; Figure 3A and Supplemental Figure 5). The number of ND MNCs generated in the presence of rTGF-β1 was reduced by 2.5-fold (Figure 3B), which was accompanied by a 7-fold reduction in the number of assayable HPCs (Figure 3C). The addition of AVID200, however, restored the number of cells generated (Figure 3B) and the levels of erythroid (BFU-E), myeloid (CFU-GM), and granulocyte, erythroid, macrophage, and megakaryocyte CFUs (CFU-GEMM) colony numbers to those observed in the presence of cytokines alone (CTR; Figure 3C). Treatment with rTGF-β1 was accompanied by activation of SMAD2 and the expression of p57^Kip2^, which was blunted by the addition of AVID200 (Figure 3D). The inhibitory effects of rTGF-β1 on normal hematopoiesis were accompanied by increased p53 and p21 protein levels, which were also downregulated by AVID200 (Figure 3D). GATA2 (48) is involved in mediating TGF-β responses in primitive hematopoietic cells by binding the regulatory region of p57^Kip2^ (49). Treatment of ND MNCs with rTGF-β1 resulted in the upregulation of GATA2, which was eliminated by AVID200 (Figure 3D). Furthermore, we observed that rTGF-β1 decreased the activation of caspase 3 (Figure 3D), suggesting that the suppressive effects of rTGF-β1 on normal cells were unrelated to activation of caspase 3 and its downstream consequences but, rather, due to the upregulation of CDKi (p21, p57^kip2^), p53, and GATA2. By contrast, phosphorylated pp38 was constitutively expressed in ND MNCs and its activation was not affected by either rTGF-β1 or AVID200 (Figure 3D). We also analyzed the activation of SMAD2/SMAD3 and pp38 at a single-cell level in normal whole blood. High-dimensional data generated by visualization of stochastic neighbor embedding (viSNE) revealed that rTGF-β1 induced pSMAD2/SMAD3 in progenitor cells (CD34), monocytes (CD14), and lymphocytes (CD19), which was dampened by AVID200 (Figure 3E and Supplemental Figure 6A). By contrast, pp38, a component of the noncanonical TGF-β pathway, was detected at high basal levels which were not affected by the addition of rTGF-β1 or AVID200 (Figure 3F and Supplemental Figure 6B). Similar analyses of MF MNC from 6 patients revealed a high degree of heterogeneity with regards to their response to rTGF-β1. The characteristics of the patients studied are provided in the Supplemental Table 1. Three out of six patient samples were responsive to exogenous rTGF-β1 and became quiescent after 48 hours of exposure to rTGF-β1 (12% vs 25%; *P* < 0.001; Figure 4A). In those specimens that were responsive to TGF-β1, the addition of AVID200 decreased the G0 fraction from 25% ± 2.6% to 14% ± 1.2% (*P* < 0.001; Figure 4A). By contrast, in those patient samples nonresponsive to rTGF-β, the fraction of cells in G0 and G1 remained similar to that of untreated cells, irrespective of the presence of AVID200 (Figure 4B). We screened TGF-β1 responsive and nonresponsive MF samples (Supplemental Figure 7A) and analyzed TGF-β1 levels in CM derived from those MNCs or MK cultures by ELISA. Nonresponsive TGF-β1 specimens generated CM with higher levels of TGF-β1 (Supplemental Figure 7, B and C).

We next examined the effects of AVID200 on TGF-β downstream signaling events in MNC from 6 additional patients with MF (patients’ clinical characteristics are described in Supplemental Table 2). MF cells isolated from 4 of 6 patients with MF remained responsive to the inhibitory actions of exogenous TGF-β1, by upregulating pSMAD2, GATA2, p57^Kip2^, p53, and p21, whereas the addition of AVD200 reversed these events, normalizing these levels (Figure 4C). By contrast, constitutively elevated basal levels of pSMAD2 were noted in MNC cells from the 2 other patients with MF, corresponding to MF5 and MF6 (Supplemental Table 2), MF5 and MF7 (Supplemental Table 3), and MF5 (Supplemental Table 1), and the addition of either TGF-β1 or AVID200 did not further affect these parameters (Figure 4D). Specifically, MF presented in Figure 4D corresponds to MF5 with additional *RAS* mutation. In both TGF-β responsive and nonresponsive MF cells, p38 MAPK was highly phosphorylated at baseline and was not further affected by the addition of rTGF-β1 or AVID200 (Figure 4, C and D).

Increased TGF-β has been implicated as a cause of marrow failure states in various hematological malignancies (50–52). We hypothesized that AVID200, by attenuating abnormal TGF-β1 signaling in MF cells, could reactivate normal HPCs rather than HPCs belonging to the malignant MF clones. To test this hypothesis, we incubated MNCs obtained from 8 different patients with MF *JAK2V617F^+^* with AVID200 and evaluated their ability to generate hematopoietic colonies. AVID200 treatment did not affect the total number of assayable MF colonies (Supplemental Figure 8) but reduced the number of *JAK2V617F^+^* colonies while increasing by 1.6-fold the number of WT colonies in 5 of the 8 patients with MF studied (Table 1 and Supplemental Figure 9). The clinical characteristics of patients responsive and unresponsive to AVID200 were similar (Supplemental Table 3).

### Treatment with AVID200 restored hematopoiesis and reduced fibrosis in the BM of Gata1^lo^ mice.

**Figure 5:**
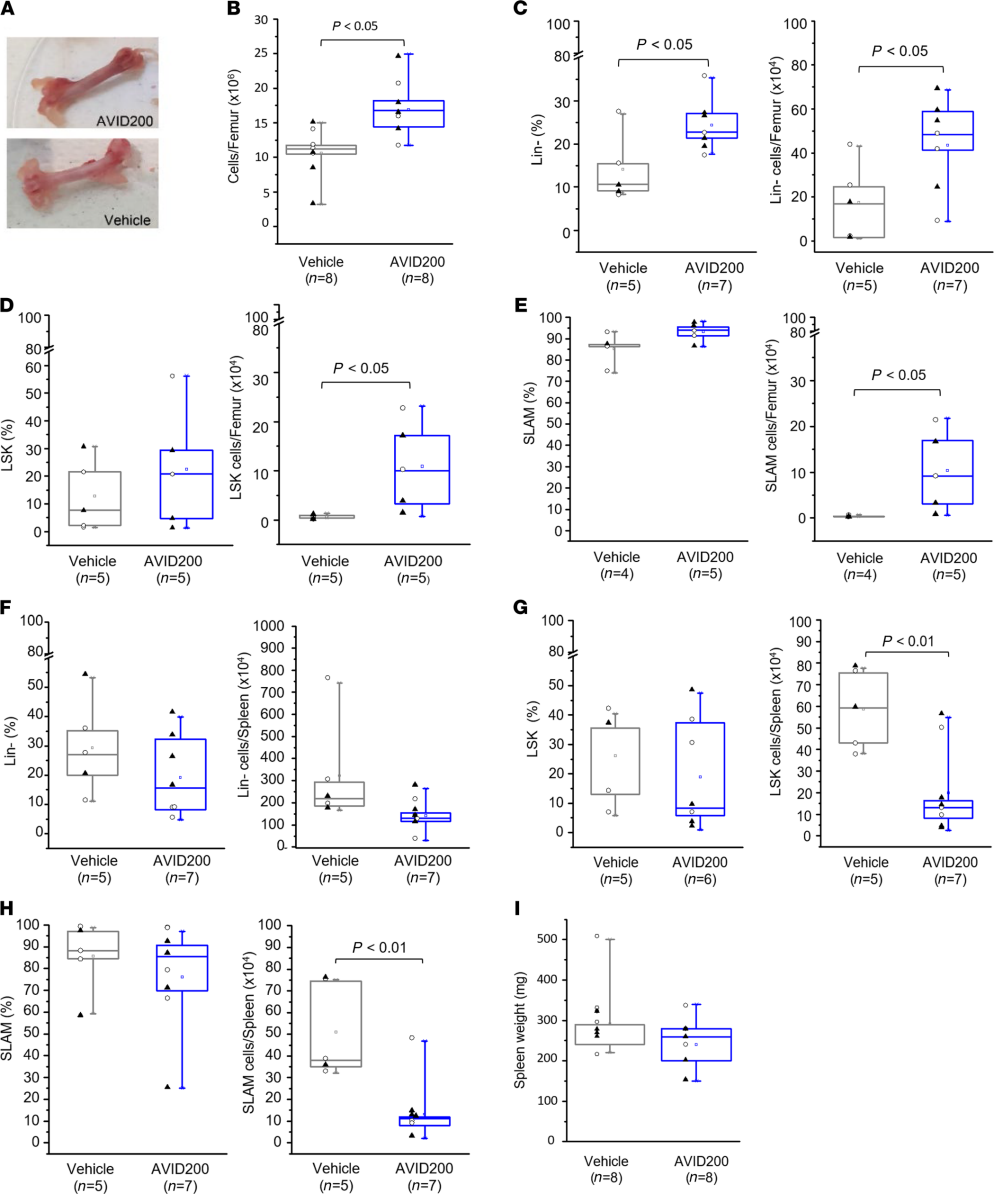
Treatment with AVID200 restores the hematopoietic stem cells in the *Gata1^lo^* mice. (**A**) Representative picture of a femur from a *Gata1^lo^* mouse treated with vehicle or AVID200 and (**B**) total number of nucleated cells per femurs. BM cell numbers are the mean ± SD of 8 mice (4 females and 4 males) per experimental group. The historic range of total cell numbers detected in femurs from untreated *Gata1^lo^* (*n* = 12) and WT (*n* = 12) littermates are 9.7 ± 0.7 and 16.2 ± 1.8 cells/femur, respectively (82). (**C**) Frequency and total number of Lin^–^, (**D**) LSK, and (**E**) SLAM cells from the femurs of *Gata*1^lo^ mice treated either with vehicle or AVID200. Values obtained in individual females (circles) and males (triangles) are also reported. (**F**) Percentage and total number of Lin, (**G**) LSK, and (**H**) SLAM cells in spleen from *Gata1^lo^* mice treated either with vehicle or with AVID200 as indicated. The gating used to define the different HSC/HPC populations is described in Supplemental Figure 10 and results are presented as box plots. (**I**) Total weight of spleens from *Gata1^lo^* mice treated with either vehicle or AVID200 was not statistically significant as indicated. The historic range of total weight of spleens from untreated *Gata1^lo^* (*n* = 12) and WT (*n* = 12) is 4.3 ± 0.45 and 1.0 ± 0.1 gr (82). The absolute number of Lin- cells was obtained by multiplying their frequency, identified by FACS, per the corresponding total number of mononuclear cells obtained from one femur. The total number of LSK was then calculated by multiplying their frequency per the corresponding total number of Lin^–^ cells and that of SLAM by multiplying their frequency per the corresponding total number of LSK. HSC, hematopoietic stem cell.

**Figure 6:**
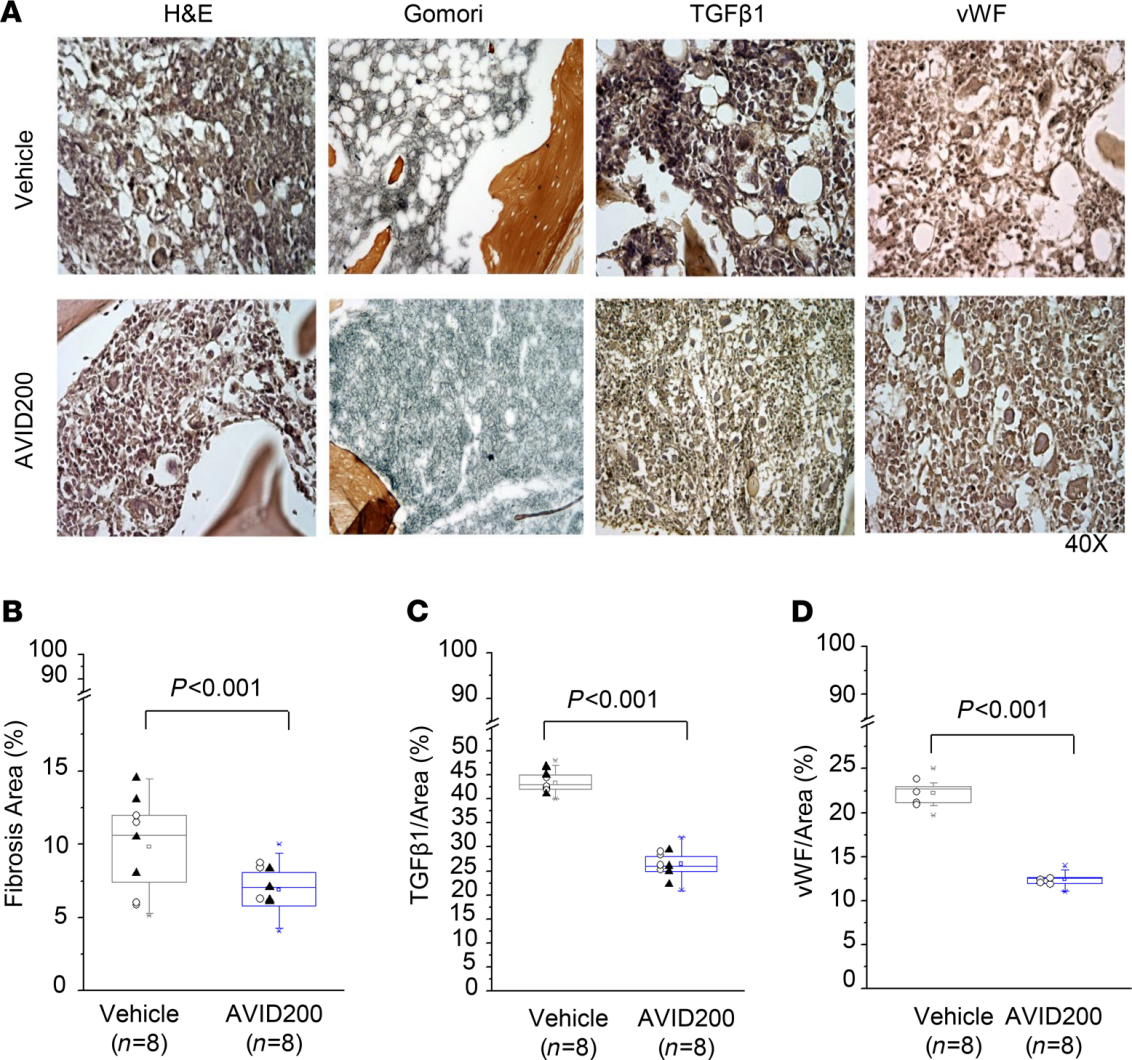
Treatment with AVID200 reduces the degree of fibrosis and TGF-β1 and vWF content in the marrow of *Gata1^lo^* mice. (**A**) H&E, Gomori silver, TGF-β1, and vWF (top panels) immunostaining of representative sections from femur of *Gata1^lo^* mice from either the vehicle-treated or AVID200-treated group. Males were sacrificed for histological analyses on day 72 (soon after the last treatment) and females on day 78 (6 days after the last treatment). (**B**) Quantification of the Gomori silver staining, and (**C**) TGF-β1, and (**D**) vWF immunostaining of the femurs from mice in the 2 groups. vWF, Von Willebrand factor.

We next evaluated the effects of AVID200 in an animal model of MF where TGF-β is known to be a mediator of BM fibrosis (53). Treatment of *Gata1^lo^* mice for 72 days with AVID200 (5 mg/Kg twice a week by i.p. injection) restored BM cellularity as reflected macroscopically by alterations in the color of the femurs and by the increased number of cells per femur (Figure 5, A and B). The frequency and numbers of the stem/progenitor cells (Lin^–^) and the total number of short-term (LSK) and long-term repopulating hematopoietic stem cells (SLAM) were increased in the femurs of mice treated with AVID200 (Figure 5, C–E, and Supplemental Figure 10). This was accompanied by a reduction in the numbers of the same subpopulation of cells in spleens (Figure 5, F–H). AVID200, however, did not induce significant changes in spleen size, weight, or peripheral blood counts (Figure 5I, Supplemental Figure 11, and Supplemental Figure 12). In addition, there was a significant reduction in the degree of fibrosis and intensity of immunochemical staining for TGF-β1 and Von Willebrand factor (vWF), a marker for increased microvessel density (54) in AVID200-treated animals (Figure 6).

## Discussion

MF is characterized by increased numbers of BM MKs that release excess amounts of proinflammatory cytokines and growth factors, which alter the hematopoietic microenvironment in a manner that supports the predominance of MF HSC/HPC (6–8). Among the MF-related proinflammatory cytokines, TGF-β plays a multitude of roles in the generation of many of the manifestations of MF, including promoting BM fibrosis, increasing marrow microvessel density, promoting the development of osteosclerosis and myeloproliferation, and suppressing normal blood cell production (9). MKs are not the only source of TGF-β in MF hematopoietic tissues; macrophages and monocytes as well as other accessory cells are also known to elaborate increased levels of this cytokine (10). Activated TGF-β1 signaling has been reported to be correlated with the degree of BM fibrosis occurring in patients with MF as well as other fibrotic disorders (55, 56). TGF-β has also been shown to contribute to the escape of tumors from immune surveillance (57, 58), which might possibly contribute to MF disease progression (59). The 3 isoforms of TGF-β (TGF-β1, TGF-β2, and TGF-β3) each regulates diverse biological functions and independently engages the specific receptor TGF-βRI/ALK5, which then undergoes dimerization with TGF-β receptor type II (TGF-βRII). Small molecule ALK5 inhibitors including SB431542 and galunisertib have been evaluated for their ability to block TGF-β signaling and reverse the fibrotic MSC phenotype and restore the marrow microenvironment in several animal models of MF (41). Furthermore, TGF-β blockade achieved with ALK5 inhibitor treatment enhances antitumor activity in several immune-competent murine solid tumor models by reversing TGF-β–mediated suppression of T cell proliferation (60). Due to these potential therapeutic effects, ALK5 inhibitors have been identified as a target for the development of therapeutic agents not only for patients with MF but also for patients with numerous solid and blood cancers.

The development of agents that block ALK5 has been hampered by concerns about potential unacceptable toxicity associated with the chronic administration of such an agent. TGF-β signaling plays an important role in the fetal development of cardiovascular organs and in the repair mechanisms of the heart (61). In particular, TGF-β2 promotes valve remodeling and differentiation by inducing matrix organization and suppressing cushion mesenchyme differentiation into cartilage during cardiac development (61). To avoid the potential adverse cardiac events associated with pan TGF-β blockade, we chose to evaluate a more selective approach.

In spite of these difficulties, ALK5 inhibitor therapy has led to promising results in patients with both solid tumors and hematological malignancies. Most relevant to their use in MF was the report of Santini and coworkers who treated patients with myelodysplastic disorders (MDS) and anemia (hemoglobin level < 10 g/dl) with galunisertib (62). Although MDS is only infrequently associated with BM fibrosis, preclinical studies indicated that TGF-β is activated in MDS stem and progenitor, resulting in the suppression of normal hematopoiesis (62). Galunisertib therapy was associated with an acceptable toxicity profile and 43% of the 41 patients treated had an improvement in their degree of anemia. Other agents that affect the TGF-β superfamily members such as sotatercept and luspatercept have also been explored to correct anemia in patients with MDS and MF (63–65). Luspatercept has been approved for use in patients with MDS for this indication and clinical trials in patients with MF are ongoing at this time (66). Sotatercept and luspatercept are, however, ALK1 rather than ALK5 inhibitors and target TGF-β signaling pathways mediated by GDF11 and activin ligands rather than TGF-β. The further clinical development of ALK5 blockade has been delayed due to their potential for promoting cardiac adverse effects. The selective effects of AVID200 on TGF-β1/TGF-β3 but not TGF-β2 represents a possible strategy to avoid the potential cardiac toxicity associated with ALK5 inhibitor therapy.

The autocrine and paracrine effects of TGF-β on tumor cells and on the tumor microenvironment have been reported to have both positive and negative effects on cancer development. Accordingly, TGF-β signaling pathway has been considered to have the potential to be both a tumor suppressor and a promoter of tumor progression and invasion (67, 68). Clinical trials of ALK5 inhibitor therapy have not provided evidence that TGF-β antagonist therapy promotes tumor progression. Furthermore, Mascarenhas and coworkers performed a phase 1 clinical trial with an anti-TGF-β antibody (GC1008) that enrolled 3 subjects before it was terminated (69). Red blood cell transfusion independence was achieved in 1 of the 3 treated subjects and 12 cycles of treatment were administered without evidence of disease progression or leukemic transformation.

We have utilized the *Gata1^lo^* mouse model of MF to further evaluate the therapeutic efficacy of AVID200. Although this MF model lacks an MPN driver mutation, the predictable progression of BM fibrosis, splenomegaly, and BM failure closely resembles the disease course of MF in humans. Previously, the inhibition of TGF-β1 signaling with the ALK5 inhibitor *SB431542* in the *Gata1^lo^* mouse was shown to restore hematopoiesis and MK development, reduce marrow fibrosis, osteogenesis, microvessel density, and the degree of extramedullary hematopoiesis (53). AVID200 therapy was also capable of reducing BM fibrosis; microvessel density, as measured by vWF immunostaining; and TGF-β in G*ata1^lo^*. We believe it is unlikely that reduced TGF-β1 content results from the TGF-β trap masking the epitope recognized by the TGF-β antibody, because similar results were observed in females who were analyzed 6 days after the last injection of AVID200. The reduction of marrow TGF-β associated with AVID200 therapy was not unanticipated because TGF-β is known to promote the production of additional levels of TGF-β (70, 71). Similarly, TGF-β and MF-MK CM increased the proliferation of human MSCs and the expression of *COL1A1*, two events associated with the MF microenvironmental abnormalities. The ability of AVID200 to dampen the actions of TGF-β in these two experimental systems suggest that this protein trap is a strong candidate for correcting MF microenvironmental abnormalities in MF.

**Figure 7:**
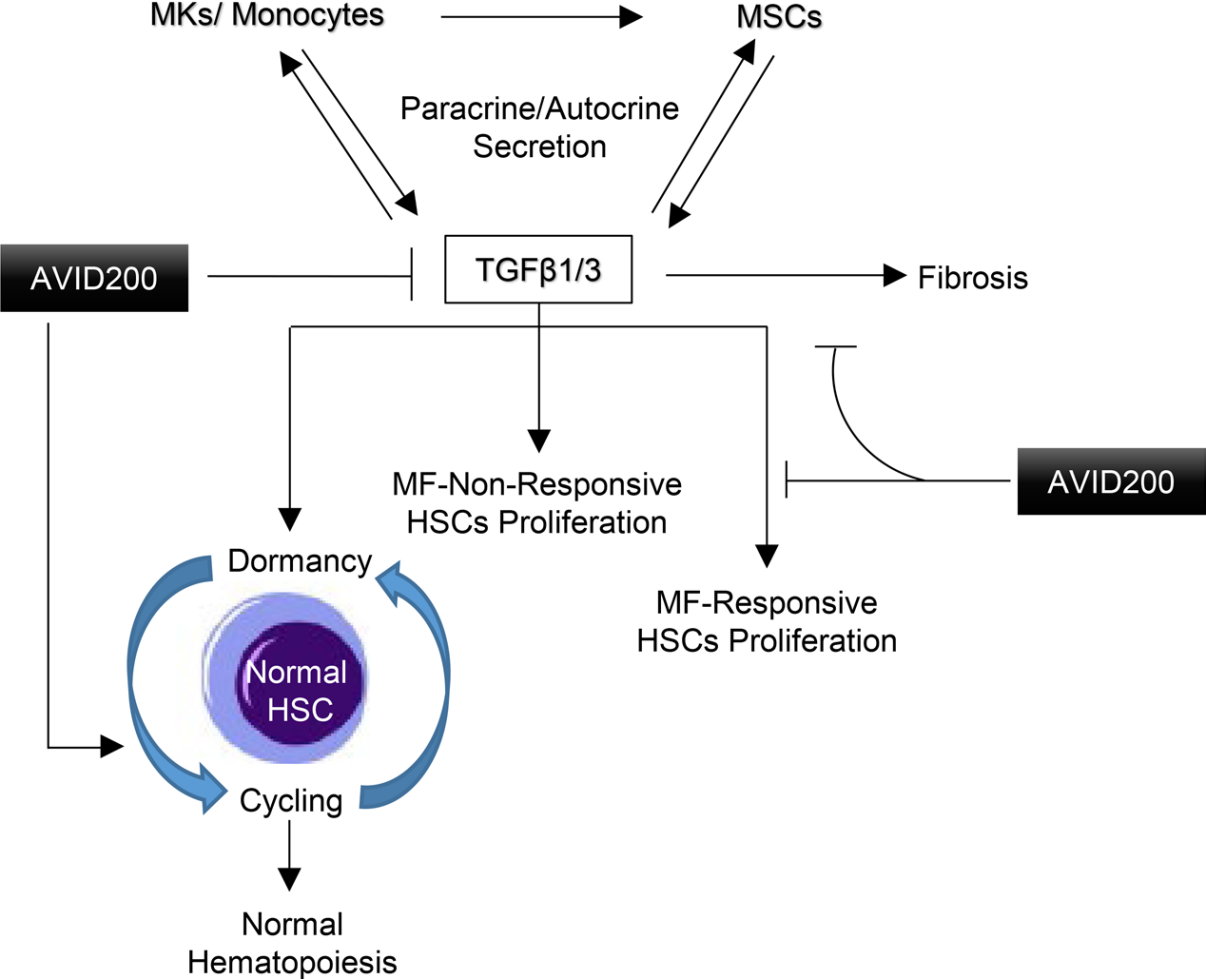
Schematic model showing the molecular mechanisms underlying the effects of AVID200 on TGF-β signaling in MF and ND cells. Excessive TGF-β secretion by MF-MKs, monocytes, and MSCs contribute to BM fibrosis and maintain the normal HSC/HPC clones in a quiescent state. AVID200, a potent antibody-like TGF-β1/TGF-β3 trap, has the potential to (a) neutralize the effects of both the autocrine and paracrine production of TGF-β1 by MKs, monocytes, and MSCs and (b) prevent TGF-β1/TGF-β3 ligand receptor interactions, which precludes the activation of pSMAD2, GATA2, and CDKi, thus allowing normal HSC/HPC from MF responsive to exit quiescence and restore normal hematopoiesis. MF, myelofibrosis; MK, megakaryocyte; ND, normal donor; MSC, mesenchymal stromal cell.

MF MNCs have a wide range of responses to TGF-β and AVID200. MNCs obtained from 50% of patients with MF remained responsive to the inhibitory effects of TGF-β1 by inhibiting MNC cycling, hematopoietic colony formation, and upregulating CDKi. Similar to ND MNCs, these effects of TGF-β1 were reversed by AVID200, thereby decreasing TGF-β signaling and restoring normal hematopoiesis by returning p53 to the required basal levels. In TGF-β1–responsive MF, the addition of rTGF-β1 had a suppressive effect on normal hematopoiesis by enhancing the fraction of cells in G0 phase. In healthy donors and in responsive MF specimens, rTGF-β1 upregulated CDKi and increased the expression of pSMAD2 and p53. P53 is known to physically interact with SMAD2 and promote the activation of multiple TGF-β target genes including p21 (72). Addition of AVID200 attenuated the suppressive effect of TGF-β signaling, allowing HSC/HPCs to exit quiescence and restore normal hematopoiesis (Figure 7). The remainder of the MF MNCs were insensitive to the actions of TGF-β1, and the levels of molecular downstream targets of TGF-β were not affected by AVID200. TGF-β unresponsive MF cells resided more in G1 phase and less in G0 phase, supporting the predominance of proliferating *JAK2V617F+* clones. The MNCs obtained from unresponsive patients were characterized by the presence of higher levels of TGF-β in CM. One of the MF samples studied had a high *JAK2V617F* VAF and an additional *RAS* mutation; AVID200 treatment of this particular specimen did not attenuate endogenous TGF-β signaling and did not allow WT clones to predominate. It is possible that *RAS* mutation may have antedated *JAK2V617F* and that the excessive TGF-β1 by altering the hematopoietic microenvironment supports the predominance of MF mutated clones. Clearly there must be underlying genetic and epigenetic changes that contribute to the altered TGF-β response. For example, in squamous carcinoma cells, oncogenic RAS signaling was shown to upregulate TGF-β production (73) and induce pSMAD2 levels (74). Moreover, recently HSPCs from *JAK2V617F* have been reported to be characterized by a high degree of heterogeneity such as different genetic subclones with distinct transcriptional signatures and subfraction of MKPs transcriptionally similar to healthy donor MKPs that can account in MF clonal evolution and therapy response (75).

These findings of resistance of MF cells have been previously reported by Ceglia et al. (76). This is in contrast to the data generated using *Gata1^lo^* mouse model, where treatment with AVID200 led to an increase in BM HSC/HPCs but a reduction in splenic HSCs/HPCs. These effects of AVID200 on *Gata1^lo^* and absence of TGF-β insensitivity in the *Gata1^lo^* mouse might reflect the absence of MPN driver mutations in this MF mouse model or an effect of the drug on HSC/HPC trafficking between the spleen and the marrow. It remains possible that the resistance to TGF-β observed in the MNCs of some patients could be the consequence of elevated levels of TGF-β1 ligand, mutations or functional inactivation of TGF-β receptors or downstream events in SMAD signaling. Such mutations or epigenetic events have been previously documented in several solid tumors and have been proposed to account for the escape of tumors from the anticipated effects of TGF-β (77). Further studies are clearly merited in defining the mechanisms that account for TGF-β resistance in this subpopulation of patients and whether it is predictive of resistance to TGF-β blockade therapy.

Ceglia and coworkers suggested that ALK5 inhibitor therapy might be useful to treat patients with MF by promoting the proliferation of normal cells that remained responsive to TGF-β, thereby allowing them to predominate at the expense of the MF cells that were insensitive to the actions of this cytokine (76). We tested this hypothesis by incubating MF MNCs in both the presence of AVID200 and the absence of AVID200 and tracking the numbers of *JAK2WT* and mutated hematopoietic colonies. MNCs were used in the studies because they contained not only HPCs but also more differentiated cells including MKs, monocytes, and accessory cells that have the potential to elaborate TGF-β. We did not have sufficient numbers of cells from these same patients to determine whether they were sensitive or insensitive to either TGF-β and/or AVID200 treatment as determined by their levels of quiescence or the expression of molecular events downstream of TGF-β. Overall, AVID200 was effective in increasing the absolute numbers of *JAK2WT* colonies by 1.6-fold in 5 of the 8 patient samples studied, with a concomitant decrease in the numbers of *JAK2V617F^+^* colonies. These findings indicate that TGF-β blockade with AVID200 affected both WT and mutated HPCs. The findings in this study are in agreement with those previously published (30, 78), which indicate that TGF-β played a pivotal role in the development of a disordered microenvironment as well as dysfunctional hematopoiesis in MF. Based upon our data, AVID200 appeared to be a candidate for reversing the BM microenvironmental abnormalities in most patients with MF because BM MSCs were not affected by MPN driver mutations. The effects of AVID200 on MF hematopoiesis were more patient-specific based upon differences in hematopoietic cell production and response to TGF-β.

## Methods

Further information and complete unedited blots can be found in Supplemental Methods.

### Human subjects.

Primary ND specimens were purchased from a commercial source (AllCells, LLC), whereas MF MNCs were provided by the Tisch Cancer Institute Hematological Malignancies Tissue Bank.

### Short-term ex vivo culture of MNCs.

MNCs obtained from NDs or patients with MF (10^6^ cells/mL) were cultured in Stemline (R)II Hematopoietic Stem (MilliporeSigma) supplemented with 100 ng/mL stem cell factor (SCF), 25 ng/mL IL-3, 50 ng/mL TPO and FLT3 (R&D) at 37°C 5% CO_2_. The cultures were replenished with fresh cytokines every 3 days and exposed to 10 ng/mL of rTGF-β1 alone or in combination with 50 nM of AVID200 for 6 days.

### Proliferation assay.

Human ND MSCs were cultured in IMDM (Thermo Fisher Scientific) containing 10% FBS, 2 mM L-glutamine, 100 U/mL of penicillin, and 100 μg/mL streptomycin. Briefly, cells were seeded into 96-well tissue culture plates (Corning Inc) at 2–5 × 10^3^/well, in 100 μl medium overnight in free IMDM. The following day, the supernatant was discarded and replaced by media supplemented with 1 ng/mL of rTGF-β1 with or without AVID200 (0.1, 10, 50 nM). CM, previously harvested from MK cultures after 11 days of culture, was concentrated 4-fold by Centricon Plus-20 tubes (MilliporeSigma), then resuspended 1:1 with IMDM (corresponding to 1 ng/mL of TGF-β) and added to MSCs alone or in combination with AVID200. CM was also incubated with and without AVID200 at 50 nM concentration for 30 minutes at room temperature before addition to the MSC cultures. After 48 and 72 hours, 20 μL MTS Luminescent Cell Viability Assay reagent (Promega) was added to each well and the plates were incubated for 1–4 hours at 37 °C. The absorbance was measured at 490 nm using a plate reader (Spectra Max M5, Molecular Device LLC).

### ELISA assay.

TGF-β1, TGF-β2, and TGF-β3 levels were quantitated using commercially available ELISA kits (DB100B, DB250, DY008, R&D Systems). Briefly, supernatants from ND or MF MNC MKs were processed and the levels of TGF-β were determined according to the manufacturer’s instructions. The colorimetric reaction was quantitated by using an absorbance multiplate reader (Spectra Max M5, Molecular Device LLC) set at 450 and 570 nm wavelengths.

### Western blotting.

Whole cell extracts were prepared from ND-MK and MF-MK, MNC, and normal MSC cultures, in NP40 buffer plus proteases and phosphatase inhibitors (Roche). Total proteins (30–50 μg) were separated on SDS-PAGE gels and transferred to nitrocellulose membranes. Transferred proteins were detected first with primary antibodies pSMAD2 (3108), SMAD2/SMAD3 (3102), pJNK (4668), JNK (9258), pSTAT3 (9145), STAT3 (9139), p53 (9282), GATA2 (4595), p21 (2947; Cell Signaling); GAPDH (CB1001, Calbiochem); p57^Kip2^ (ab119989, Abcam); and then with appropriate horseradish peroxidase–coupled secondary antibodies (Calbiochem). Immune complexes were detected with an enhanced chemiluminescence kit (Amersham) or LUM-LIGHT^Plus^ (Roche).

### qRT-PCR analysis.

Total RNA was extracted from cultured MSCs by RNeasy Mini Kit (QIAGEN) and converted in cDNA by Omniscript RT kit (QIAGEN) and added to TaqMan PCR mix (Applied Biosystem) in a final volume of 25 μl containing forward and reverse primers (*COL1A1* Hs00164004-m1 and *GAPDH* H03929097-g1; Applied Biosystem). Data were normalized to *GAPDH* by relative quantification using ΔΔCt method.

### HPC assays.

MNCs obtained from NDs or patients with MF were resuspended at 25 × 10^4^/mL in Stemline (R) II Hematopoietic Stem (MilliporeSigma) media in the presence/absence of 10 ng/mL rTGF-β1 or 50 nM AVID200 alone or in combination, and preincubated for 1 hour at 37°C/ 5% CO_2_ before being mixed in 3 mL of Methocult H4236 (Stem Cell Technology) supplemented with 100 ng/mL of SCF; 25 ng/mL of IL-3; 50 ng/mL of FLT3, TPO, GM-CSF, and IL-6; 1 U/mL of EPO; and 50 nM of AVID200. After 14 days of incubation at 37°C in 5% CO_2,_ colonies were enumerated.

### Nested allele-specific PCR for JAK2V617F.

MF colonies incubated with or without AVID200 were enumerated, plucked, and genotyped for *JAK2V617F* by nested allele-specific PCR. Genomic DNA was isolated from randomly plucked 30 to 50 colonies using the Extract-N-Amp Blood PCR Kit (MilliporeSigma). The final PCR products were analyzed in 2% agarose gels. Colonies were classified as homozygous for *JAK2V617F* if only the 279-bp band was detected, heterozygous colonies if both a 279-bp and 229-bp bands were observed, and WT if only a 229-bp band was detected.

### Mass cytometry (CyTOF).

Healthy human adult or cord blood (400 μl) were incubated with 10 or 100 ng/mL rTGF-β1 and 50 or 100 nM AVID200 at 37°C for 30 minutes. After incubation, the blood was fixed with Smart Tube Proteomic stabilizer 1 buffer (SMART TUBE) as per the manufacturer’s instructions and stored at –80°C until staining was performed. To obtain cells for CyTOF staining, stabilized blood samples were thawed in a 15°C water bath and RBCs were lysed with Thaw Lyse buffer (SMART TUBE) according to the manufacturer’s instructions. Cells were washed with cell staining buffer (PBS with 0.2% BSA and 0.02% NaN3) and stained with the CyTOF antibody panel (see Supplemental Table 4). Initially, the cells were stained with an antibody cocktail comprised of only cell surface markers for 30 minutes on ice. Surface stained cells were then washed and fixed with 1.6% formaldehyde for 10 minutes. Fixed cells were then methanol permeabilized and further stained for 30 minutes on ice with PE-anti-pSMAD2/3 (clone O72-670, BD Biosciences) followed by a 30-minute staining with an antibody cocktail comprising of phospho-specific antibodies and anti-PE (Supplemental Table 4). After staining, cells were washed and fixed in freshly diluted 2.4% formaldehyde containing 0.08% saponin, 125 nM intercalator-Ir (Fluidigm), and 300 nM OsO4 (ACROS Organics) for 30 minutes. Samples were stored at 4°C in cell staining buffer containing 125 nM intercalator-Ir until acquisition. Prior to acquisition samples were washed with CAS buffer (Fluidigm) and resuspended in CAS buffer containing EQ normalization beads (Fluidigm) and acquired on a CyTOF2 (Fluidigm). The data were normalized using bead-based normalization in the CyTOF software and exported to Cytobank software for analysis. Data were first gated to exclude beads and doublets and then manual gating was performed using canonical surface markers to identify different cell types. The gated cell population are indicated in the Supplemental Table 5. Heatmaps representing median staining intensity were generated with Cytobank software.

### Treatment of Gata1^lo^ mice with AVID200.

*Gata1^lo^* mice were bred at the animal facility of Istituto Superiore di Sanità (Rome, Italy) as described (28, 53). Littermates were genotyped at birth by PCR and those found not to carry the mutation were used as WT controls. *Gata1^lo^* mice (10–12 months old) were weighted and randomly divided into 2 groups (4 males and 4 females per group) and treated with either vehicle (PBS) or AVID200 (5 mg/Kg twice a week by i.p. injection). We conducted 2 sequential studies. In the first experiment, mice were treated for 42 days with either AVID200 (6 mice, 3 females and 3 males) or vehicle (2 females). In the second experiment, mice (8 mice, 4 females, and 4 males in each group) were treated for 72 days. On days 42, 72 (males), or 78 (females), mice were weighted, bled for blood count determinations, and euthanized for organ histopathology observations. The treatment was well tolerated during both experiments and only 2 deaths were recorded (1 in the vehicle and the other in the AVID200 group) in the first experiments.

### Flow cytometry.

The entire mouse femur (bone and medulla) and spleen were suspended in media, and the number of cells were enumerated, washed once with PBS-5% (v/v) FBS, and resuspended in the same solution containing a cocktail of the following antibodies: APC-CD117, APC-Cy7-Sca1, PE-Cy7-CD150, biotin-labeled anti–mouse CD48, and biotin-labeled anti-lineage antibodies. All antibodies were purchased from BD Pharmingen. After 30 minutes of incubation on ice, cells were washed once and stained with streptavidin-PE-Cy5, and cell fluorescence was analyzed with a Gallios analyzer (Beckman Coulter). HPCs were defined as lineage negative (Lin^–^) cells. The total hematopoietic stem cell populations were defined as LSK (Lin^–^/CD48^neg^/c-Kit^pos^/Sca-1^pos^) and long-term repopulating hematopoietic stem cells were defined by the SLAM phenotype (Lin^–^/CD48^neg^/c-Kit^pos^/Sca-1^pos^/CD150^pos^) as previously described (79, 80). Nonspecific signals and dead cells were excluded by appropriate fluorochrome-conjugated isotype and propidium iodide staining, respectively. Results were analyzed with the Kaluza analysis version 2.1 (Beckman Coulter).

### Histology.

Femurs were fixed in 10% (v/v) phosphate-buffered formalin and paraffin-embedded and cut into consecutive 2.5–3 μm sections that were then stained by either H&E or Gomori silver (MicroStain MicroKit) staining or immunostaining with an anti-TGF-β1 antibody (sc-146, Santa Cruz Biotechnology) or anti-Von Willebrand factor (AB 11713, Abcam) revealed with avidin-biotin immunoperoxidase (Vectastain Elite ABC Kit; Vector Laboratories) and HISTO-STAT Peroxidase Kits (Innovex Biosciences), as previously described (53). Images were acquired with a ZEISS Axioskop light microscope equipped with a Coolsnap Videocamera. The signals of interest were quantified with the freeware software NIH ImageJ (version 1.29); positive staining was defined evaluating threshold values in a nonfibrotic area in each sample. Results are expressed as percentage of area stained above threshold with respect to the total area analyzed by the program, as previously described (81). Quantification of fibrosis and TGF-β1 content were done on at least 3 different areas for consecutive individual mouse femur sections.

### Statistics.

Because there was no statistical difference among the results obtained using male and female mice in both groups (see the distribution of the data obtained from individual mice presented in Figure 5), the data from male and female mice were pooled and presented either as mean ± SD or as median, 25%–75% IQR range and maximum and minimum value, as specified in the legend of the figures. Statistical analysis was performed by 1-way ANOVA using Origin 3.5 software for Windows (Microcal Software; http://www.originlab.com).

### Study approval.

Experiments were performed according to the protocol number 4f9l2015-PR (Risp. a DGSAF-A 21303), approved by the Italian Ministry of Health on May 22, 2015, and were conducted according to the European Directive for animal experiments 86/609/EEC. All subjects provided written informed consent according to guidelines approved by the IRB of the Icahn School of Medicine at Mount Sinai in accordance with the Declaration of Helsinki’s for studies involving human subjects (approval HS number 17-01337).

## Author contributions

LV, CIR, and RH conceived the study. CC processed patient samples. LV, BU, JFD, GT, MZ, FM, and PV performed experiments and analyzed data. AHR supervised the CyTOF data. MOM provided AVID200 and interpreted the data. ARM supervised and interpreted the mouse data. LV and RH interpreted the data and wrote the manuscript. JM and RAM enrolled and consented patients for blood samples collection. All authors discussed the results and commented on the manuscript.

## Supplementary Material

Supplemental data

## Figures and Tables

**Table 1:**
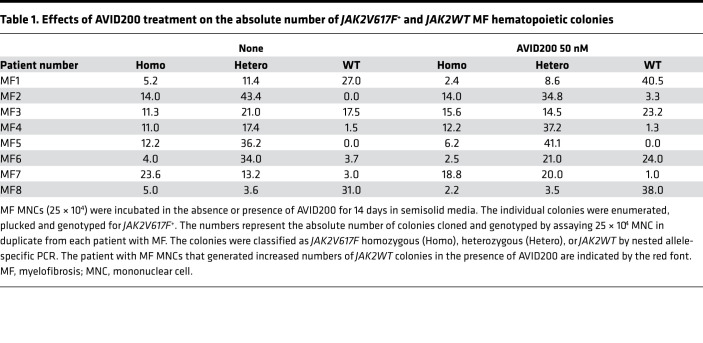
Effects of AVID200 treatment on the absolute number of *JAK2V617F*^+^ and *JAK2WT* MF hematopoietic colonies

## Version 1 08/12/2021

In-Press Preview

## Version 2 09/22/2021

Electronic publication
