# Supplementary material for: TGF-β1 protein trap AVID200 beneficially affects hematopoiesis and bone marrow fibrosis in myelofibrosis
Source: JCI Insight. 2021 Sep 22;6(18):e145651. doi: 10.1172/jci.insight.145651 (PMC8492354; doi:10.1172/jci.insight.145651)
Supplement: Supplemental data [file jciinsight-6-145651-s227.pdf]

TGFβ1 Protein Trap AVID200 Beneficially Affects Hematopoiesis and Bone Marrow Fibrosis in Myelofibrosis

Lilian Varricchio[1,*], Camelia Iancu-Rubin[1,2], Bhaskar Upadhyaya[3], Maria Zingariello[4], Fabrizio Martelli[5], Paola Verachi[6], Cara Clementelli[1], Jean-Francois Denis[7], Adeeb Rahman[3], Gilles Tremblay[7], John Mascarenhas[1], Ruben A. Mesa[8], Maureen O'Connor-McCourt[7], Anna Rita Migliaccio[6], Ronald Hoffman[1*]

1 Tisch Cancer Institute, Icahn School of Medicine at Mount Sinai, New York, New York, USA. **2** Department of Pathology, Molecular and Cell-Based Medicine, Icahn School of Medicine at Mount Sinai, New York, New York, USA. **3** Human Immune Monitoring Core, Icahn School of Medicine at Mount Sinai, New York, New York, USA. **4** Unit of Microscopic and Ultrastructural Anatomy, Department of Medicine, University Campus Bio-Medico, Rome, Italy. **5** National Center for Drug Research and Evaluation, Istituto Superiore di Sanità, Rome, Italy. **6** Biomedical and Neuromotorial Sciences, Alma Mater University, Bologna, Italy. **7** Forbius, 750 Boulevard, Saint-Laurent, Montreal, Quebec, Canada. **8** Mays Cancer Center, 7979 Wurzbach Road, 78006, San Antonio, TX, USA.

Corresponding authors: Ronald Hoffman, Albert and Vera G. List Professor of Medicine, Division of Hematology and Medical Oncology1 Gustave L. Levy Place Tisch Cancer Institute, Icahn School of Medicine at Mount Sinai, Box 1079, New York, New York, USA; ronald.hoffman@mssm.edu; Lilian Varricchio, Tisch Cancer Institute, Icahn School of Medicine at Mount Sinai, New York, New York, USA; lilian.varricchio@mssm.edu

SUPPLEMENTAL METHODS

Ex vivo generation human MKs and their phenotypic analysis. All MNC were prepared by standard Ficoll-Hypaque centrifugation (Amersham Pharmacia Biotech, Uppsala, Sweden). Ex vivo generation of MKs was performed as previously described (1). Cytospin preparations were obtained by cytocentrifugation (Shandon, Astmoor, UK), stained with May-Grünwald-Giemsa (Sigma) and then examined by light microscopy (Magnification 40x). For flow cytometry, cells were labelled with FITC-conjugated CD41 and APC-conjugated CD42 (Becton Dickinson Biosciences, Franklin Lakes, NJ). Dead cells were identified by propidium iodide staining (Sigma). Data acquisition and analysis were performed on a Canto cytometer using DIVA software (Becton-Dickinson, Mountain View, CA).

A549/IL-11 release assay of TGFβ activity. A549 human non-small cell lung carcinoma cells were purchased from ATCC (Manassas, VA) and cultured in RPMI1640 media (Thermo Fisher Scientific, Waltham, MA, USA) containing 5% fetal bovine serum and 4 mM L-Glutamine. Briefly, A549 cells were seeded in a 96-well plates (5000 cells/well in 100 µL medium) and incubated in a humidified incubator at 37°C, 5% CO_2 overnight. The following day, the medium was aspirated and replaced by growth media containing 10 pM of either TGFβ1, TGFβ2 or TGFβ3 in the presence of increasing concentrations of AVID200 (Forbius, Austin, Texas and Montreal, CA) (range: 0.01, 0.1, 1.0, 3.0, 10, 30, 100, 300, 1000, 10000 or 100000 pM). The TGFβ ligands were pre-incubated with AVID200 for 30 min at room temperature before being added to the cells. After 24h of incubation, the CM was harvested, centrifuged and the concentration of IL-11 was determined using an ELISA according to the manufacturer’s instruction (Human IL-11

Immunoassay Quantikine ELISA Kit, Cat# D1100, R&D Systems, Inc.). All experiments were conducted in triplicate. The data was normalized to the IL-11 concentration in control wells containing only TGFβ, which was set to 100%.

Nested allele-specific primer polymerase chain reaction for *JAK2V617F*. *JAK2V617F* was detected by using a nested allele-specific primer PCR sequences: Forward 5′-GATCTCCATATTCCAGGCTTACACA-3′; reverse 5′-TATTGTTTGGGCATTGTAACCTTCT-3′, following 35 cycles of 30s at 95°C, 30s at 60°C and 30s at 72°C. PCR products from 1st step, were further amplified by nested allele-specific-primers: 5'-CCTCAGAACGTTGATGGCA-3'; 5'-ATTGCTTTCCTTTTTCACAAGA-3';

5'-AGCATTTGGTTTTAAATTATGGAGTATATG-3';

5'-GTTTTACTTACTCTCGTCTCCACAAAA-3', following 35 cycles of 95°C for 30s, 59°C for 25s, and 72°C for 25s.

Hoechst/Pyronin staining. Cells were resuspended in Stemline (R) II Hematopoietic Stem (Sigma Aldrich) media and then stained with 1 μg/mL Hoechst 33342 (Invitrogen) at 37°C for 45 minutes, followed by 1 μg/mL Pyronin Y (Sigma Aldrich) for 15 minutes. After staining the cells were washed, resuspended in MACs' buffer and analyzed with BD LSR Fortessa flow cytometer (BD Biosciences) Diva software. All gates were set based on unstained control samples.

Blood count determinations. Blood (200 μl) was collected at day 0, 30 and 72 post treatment from the retro-orbital plexus of anesthetized mice. Hematocrit (Htc), Hemoglobin (Hb), platelets (Plts) and white blood cells (WBC) counts, were evaluated by an accredited commercial provider (Plaisant Laboratory, Rome, Italy).

References

1. Mosoyan G, Kraus T, Ye F, Eng K, Crispino JD, Hoffman R, et al. Imetelstat, a telomerase inhibitor, differentially affects normal and malignant megakaryopoiesis. *Leukemia.* 2017;31(11):2458-67.

Supplemental Results

Treatment with AVID200 is well tolerated and does not alter blood cell counts. Treatment was extremely well tolerated. In the second experiment, no death was recorded over a period of 72 days of manipulation/observation. In addition, in both groups, body weight remained within normal ranges (day 0: 37+/-3.88g; day 40: AVID200 37.94+/-3.08g vs vehicle 38.56+/-3.37g; day 54: AVID200 37.38+/-4.01g versus vehicle 38.56+/-3.01g; Day 78: AVID200 36.88+/-3.01g versus vehicle 38.39+/-2.31g) and fur was slightly more luster in the AVID200 group (**Supplemental Figure 11**). Blood counts showed increases in RBC counts (from 4.07+/-1.27 at day 0 to 6.35+/-0.93 $x10^6$/uL AVID200 and 7.11+/-0.96 $x10^6$/uL vehicle, at day 72) and Hgb (from 6.21+/-2.08 at day 0 to 11.24+/-1.54, AVID200, and 12.03+/-1.51 g/dL, vehicle at day 72) both in the AVID200 and vehicle group (**Supplemental Figure 12**). We believe that these increases are the result of stress erythropoiesis activated by the anemia induced by serial phlebotomies. By contrast, ptl (from 133+/-50 at day 0 to 94+/-37, AVID200, and 105+/-28 $x10^3$/uL, vehicle, at day 72) and WBC (from 3.9+/-0.7 at day 0 to 4.6+/-1.39, AVID200, and 4+/-0.95 $x10^3$/uL, vehicle at day 72) did not significantly change with treatment (**Supplemental Figure 12**). Post-mortem necropsy examination did not reveal any grossly morphological abnormalities in the organs in both groups. Specifically, the heart and aorta of the AVID200 mice had normal appearance (data not shown).

Supplemental Table

Supplemental Table 1. Clinical characteristics of the patients from whom samples were obtained for cell cycle analyses.

PT	Diagnosis	Age of Diagnosis	Sex	JAK2 VAF	Additional Mutations	Prior Treatment	Current Treatment	In vitro Response to AVID200
MF1	PET-MF	64	M	28	SRSF2 - 7%	Anagrelide	Ruxolitinib	Yes
MF2	PMF	70	M	37	TET2 - 44%, U2AF1 - 33%	None	None	Yes
MF3	PMF	70	M	32	EZH2 - 29%, NRAS - 37%, PHF6 - 6%, RUNX1 - 15%, SETBP1 - 28%	Pembrolizumab, Pacritinib	Decitabine	Yes
MF4	PMF	71	M	48	ASXL1 - 43%, CBL - 89%, NF1 - 48%, RUNX1 - 50%, SRSF2 - 45%	None	Ruxolitinib	No
MF5	PPV-MF	48	F	85	TET2 - 48%	None	Ruxolitinib	No
MF6	PPV-MF	64	F	26.4	None	Pomalideomide, Danazol, Decitabine	Pacritinib	No

Supplemental Table 2. Clinical characteristics of the patients from whom samples were used in the analysis of the molecular targets of TGFβ.

PT	Diagnosis	Age of Diagnosis	Sex	JAK2 VAF	Additional Mutations	Prior Treatment	Current Treatment	In vitro Response to AVID200
MF1	PMF	82	Female	7	None	None	Ruxolitinib	Yes
MF2	PMF	61	Male	67	None	None	Ruxolitinib	Yes
MF3	PPV-MF	58	Male	87.4	None	Hydroxyurea	Ruxolitinib	Yes
MF4	PPV-MF	55	Male	54.2	none	Ruxolitinib	Ruxolitinib	Yes
MF5	PPV-MF	65	M	81.58	KRAS - 42%	Hydroxyurea, Anagrelide, Panobinostat	Ruxolitinib	No
MF6	PPV-MF	48	F	85	TET2 - 48%	Hydroxyurea, Anagrelide	Ruxolitinib	No

Supplemental Table 3. Clinical characteristics of the patients from whom samples were used in the analysis of the genotyping assay of MF hematopoietic colonies.

PT	Diagnosis	Age of Diagnosis	Sex	JAK2 VAF	Additional Mutations	Prior Treatment	Current Treatment	In vitro Response to AVID200
MF1	PET-MF	78	F	8.58	None	Hydroxyurea, Anagrelide	Ruxolitinib	Yes
MF2	PMF	66	M	82	ASXL1 - 48%, TET2 - 46%, U2AF1 - 49%	Ruxolitinib, CPI-0610	None	Yes
MF3	PPV-MF	75	F	n.a.	None	Hydroxyurea	Ruxolitinib	Yes
MF4	PPV-MF	65	M	93	None	Hydroxyurea, Peg-IFN	Peg-IFN	No
MF5	PPV-MF	65	M	81.58	KRAS - 42%	Hydroxyurea, Anagrelide, Panobinostat	Ruxolitinib	No
MF6	PMF	66	M	51	SF3B1 - 33%	Pembrolizumab ,Ruxolitinib	None	Yes
MF7	PPV-MF	48	F	85	TET2 - 48%	Hydroxyurea, Anagrelide	Ruxolitinib	No
MF8	PET-MF	64	M	28	SRSF2 - 7%	Hydroxyurea, Anagrelide	Ruxolitinib	Yes

Supplemental Table 4. Antibodies used for CyTOF analysis

CyTOF Panel	**Vendor**	**Catalog Number**	**Clone**
Anti-human CD45 – Y89	Fluidigm	3089003B	HI30
Anti-human CD19 – Nd142	Miltenyi	130-122-301	REA675
Anti-human CD45RA – Nd143	Miltenyi	130122-292	REA562
Anti-human CD4 – Nd145	Miltenyi	130-122-283	REA623
Anti-human CD8 – Nd146	Miltenyi	130-122-281	REA734
Anti-human CD16 – Nd148	Miltenyi	130-108-027	REA423
Anti-human CD1c – Nd150	Miltenyi	130-122-298	REA694
Anti-human CD123 – Eu151	Miltenyi	130-122-297	REA918
Anti-human CD66b – Sm152	Miltenyi	130-108-019	REA306
Anti-human CD27 – Gd155	Miltenyi	130-122-295	REA499
Anti-human pp38 – Gd156	Fluidigm	3156002A	D3F9
Anti-human pMAPKAPK2 – Tb159	Fluidigm	3159010A	27B7
Anti-human CD14 – Gd160	Biolegand	301802	M5E2
Anti-human CD56 – Dy161	Miltenyi	130-108-016	REA196
Anti-human pSMAD2/3-PE	BD Biosciences	562586	O72-670
Anti-PE – Ho165	Biolegand	408102	PE001
Anti-human CD34 – Er167	Biolegend	343502	581
Anti-human CD3 – Er168	Miltenyi	130-122-282	REA613
Anti-human CD38 – Er170	Miltenyi	130-122-288	REA671
Anti-human pERK1/2 – Yb171	Fluidigm	3171010A	D13.14.4E
Anti-human HLADR – Yb174	Miltenyi	130-122-299	REA805
Anti-human pS6 – Lu175	Fluidigm	3175009A	N7548

Supplemental Table 5. Immune cell population definitions

**Population**	**Phenotype**
CD34+	CD66b-CD34+
Neutrophils	CD34-CD66b+
Basophils	CD34-CD66b-CD14-CD19-CD3-HLADR-CD123+
CD14+ Monocytes	CD34-CD66b-HLADR+CD19-CD3-CD123-CD1c-CD16-CD14+
CD16+CD14-/low monocytes	CD34-CD66b-HLADR+CD19-CD3-CD123-CD1c-CD14-/lowCD16+
mDC	CD34-CD66b-CD14-HLADR+CD19-CD3-CD123-CD1c-CD16-
pDC	CD34-CD66b-CD14-CD19-CD3-HLADR+CD123+
CD1c+ DC	CD34-CD66b-CD14-HLADR+CD19-CD3-CD123-CD1c+
CD3 T cells	CD34-CD66b-CD14-CD16-CD1c-CD123-CD3+
CD4 T cells	CD34-CD66b-CD14-CD16-CD1c-CD123-CD3+CD8-CD4+
CD8 T cells	CD34-CD66b-CD14-CD16-CD1c-CD123-CD3+CD4-C8+
Memory CD4 T cells	CD34-CD66b-CD14-CD16-CD1c-CD123-CD3+CD8-CD4+ CD45RA-
Naive CD4 T cells	CD34-CD66b-CD14-CD16-CD1c-CD123-CD3+CD8-CD4+ CD45RA+
Memory CD8 T cells	CD34-CD66b-CD14-CD16-CD1c-CD123-CD3+CD4--CD8+CD45RA-
Naive and EMRA CD8 T cells	CD34-CD66b-CD14-CD16-CD1c-CD123-CD3+CD4-CD8+ CD45RA+
B cells	CD34-CD66b-CD14-CD3-CD19+
NK cells CD56lowCD16hi	CD34-CD66b-CD14-HLADR-CD19-CD3-CD123-CD1c-CD56lowCD16hi
NK cells CD56hiCD16low	CD34-CD66b-CD14-HLADR-CD19-CD3-CD123-CD1c-CD56hiCD16low
NK cells CD56lowCD16-	CD34-CD66b-CD14-HLADR-CD19-CD3-CD123-CD1c-CD56lowCD16-

Supplemental Figures

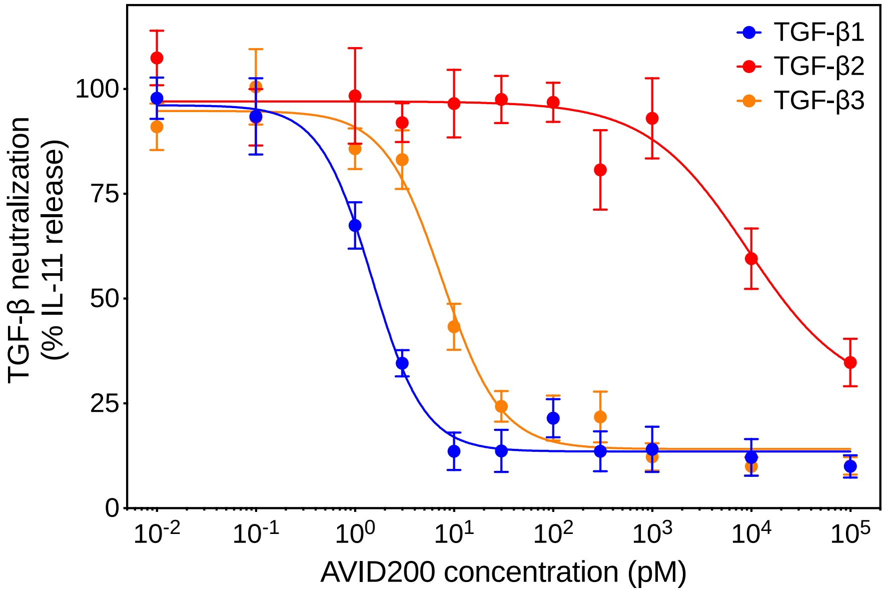

Supplemental Figure 1. AVID200 selectively neutralizes the activity of TGFβ1 and TGFβ3. Neutralization of TGFβ1, TGFβ2, or TGFβ3 activity was measured in the presence of increasing AVID200 concentrations as indicated. A549 cells release IL-11 in response to TGFβ, which was measured using an ELISA. The data is representative of at least four experiments and the results are presented as mean percentage ± SEM. The figure was generated using Prism 8 and IC50 values were calculated using a variable slope, four parameters non-linear regression.

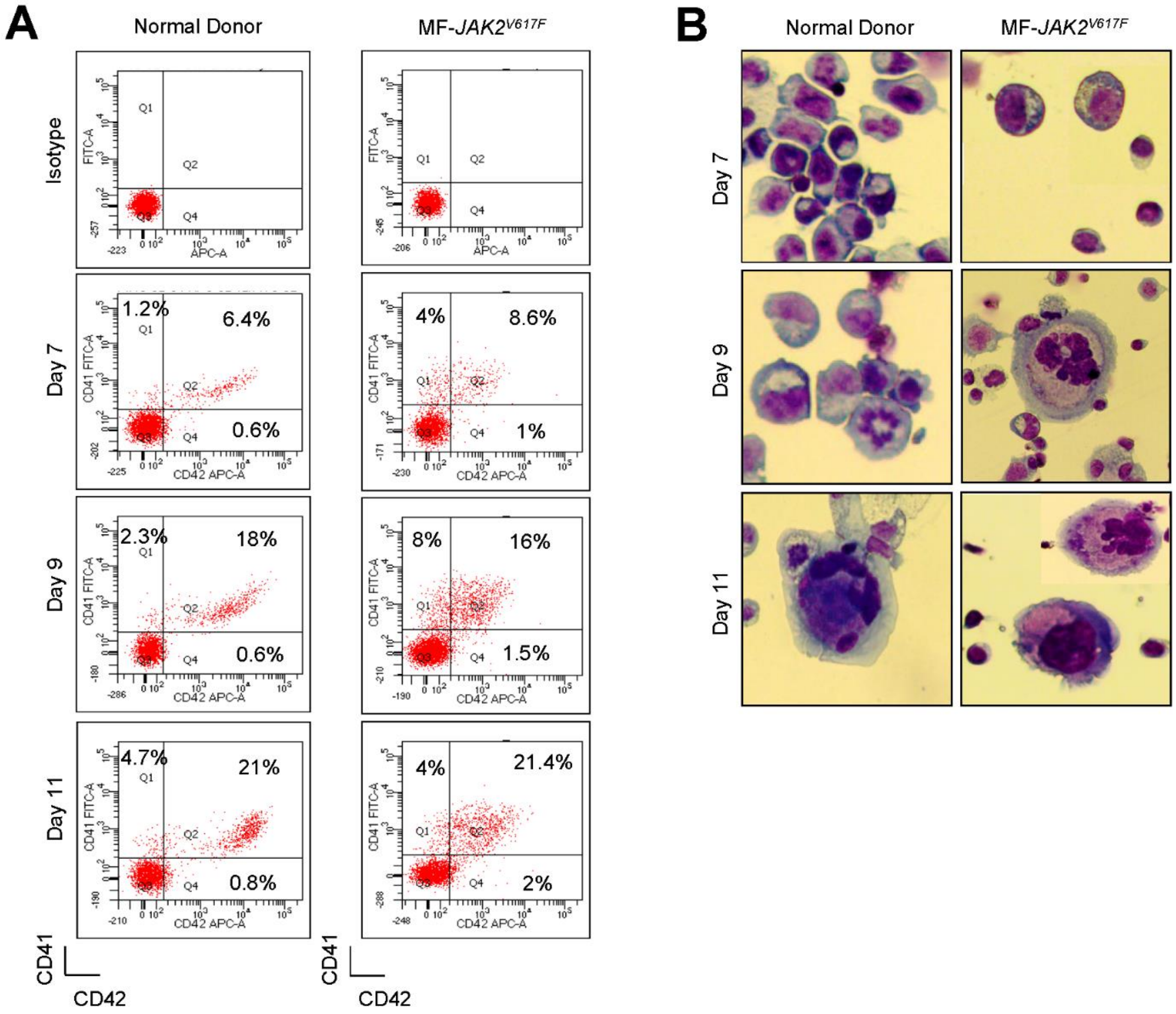

Supplemental Figure 2. MK cultures generated by ND and MF MNCs. A) Representative flow cytometry dot plot of cells generated by ND or MF MNCs stained with anti CD41 and CD42 monoclonal antibodies. **B)** Representative cytospins showing the morphological appearance of ND and MF MKs generated from corresponding MNCs after 7-11 days of culture.

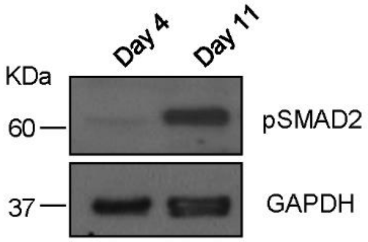

Supplemental Figure 3. pSMAD2 expression during MK development. Expression of pSMAD2 during the proliferative (day 4) and differentiative (day 11) phase of MK cultures. GAPDH was loaded as an internal control.

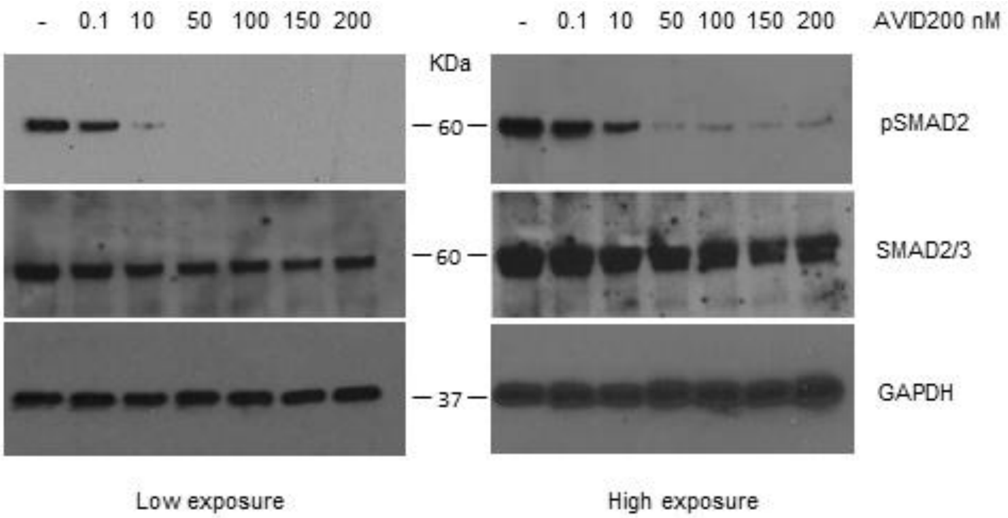

Supplemental Figure 4. Increasing concentrations of AVID200 inhibit pSMAD2 expression. MF-MK cultures (from day 9 of differentiation) were exposed to increasing concentrations of AVID200 for 48 hours. Western Blot for pSMAD2 and total SMAD2/3. GAPDH was used as an internal loading control. Low and high exposure by Lumi-Light^{PLUS} substrate.

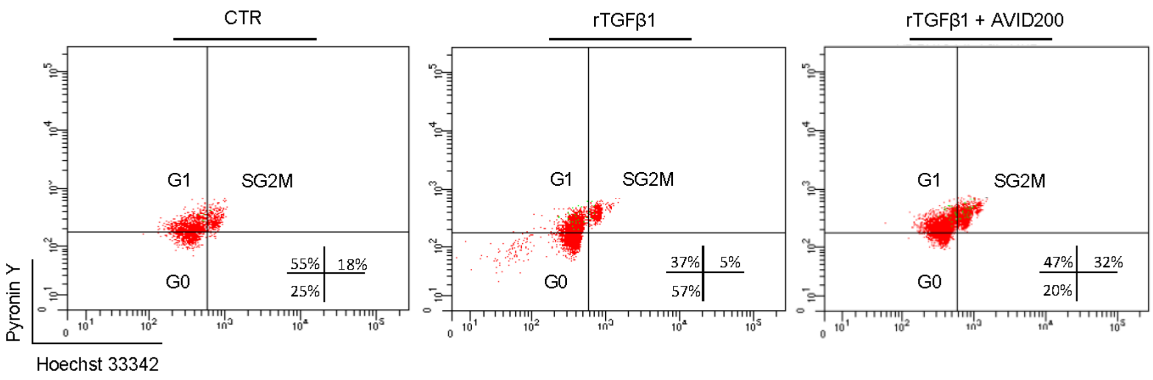

Supplemental Figure 5. Representative FACS plot of MNCs stained with Hoechst 33342 and Pyronin Y. Cells in the lower left quadrant represent cells in G0, those in upper left quadrant represent cells in G1 phase, and those in upper right quadrant represent cells in SG2M. The percentages indicate the fraction of cells in G0, G1, SG2M in untreated cells (CTR), cells treated with rTGFβ1 alone or in combination with AVID200 for 48 hours, as evaluated by staining with, Hoechst 33342 and Pyronin Y (n=3 biological replicates).

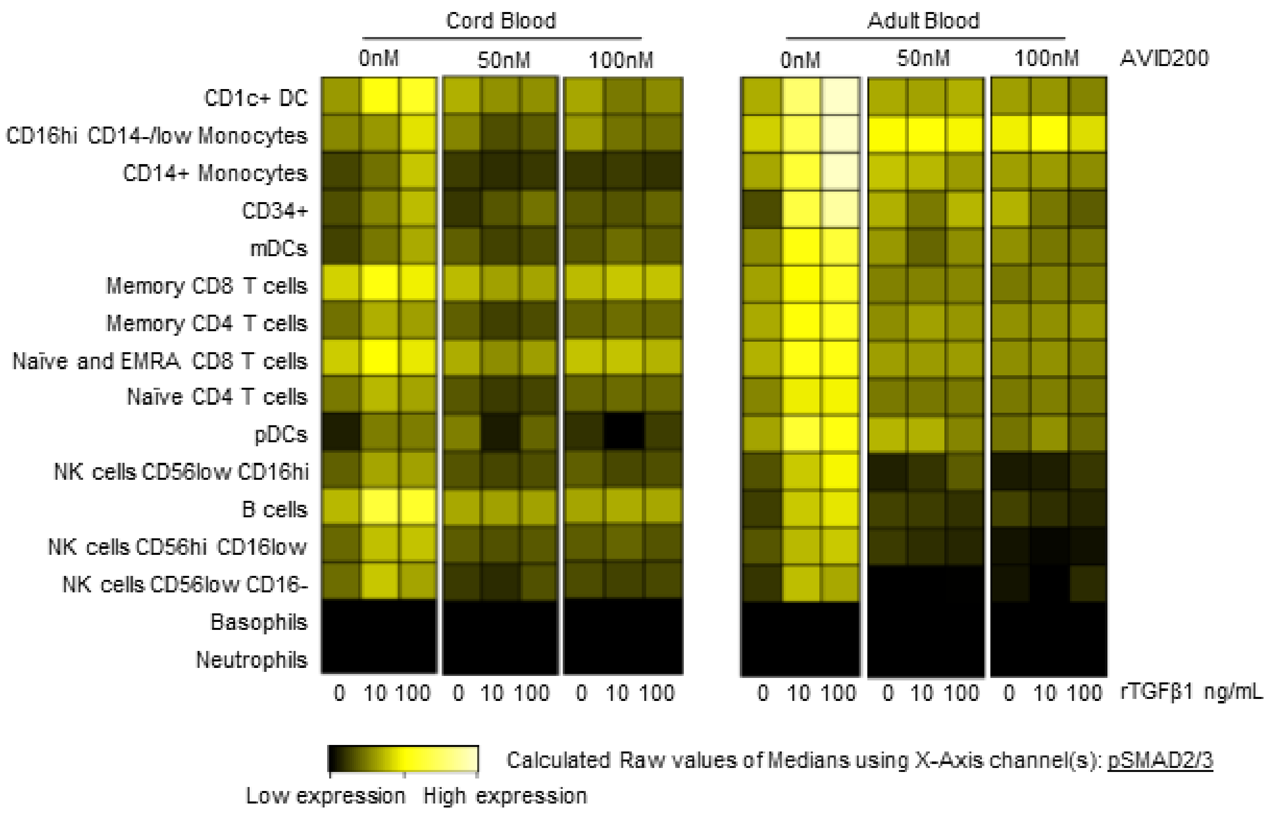

Supplemental Figure 6A. CyTOF analysis reveals that AVID200 inhibits the effect of rTGFβ1 on pSMAD2/3 levels. ViSNE analysis for pSMAD2/3 expression levels in different gated cell populations from healthy donor whole blood (cord and adult blood) treated with rTGFβ1 and AVID200 alone or in combinations at increasing concentrations.

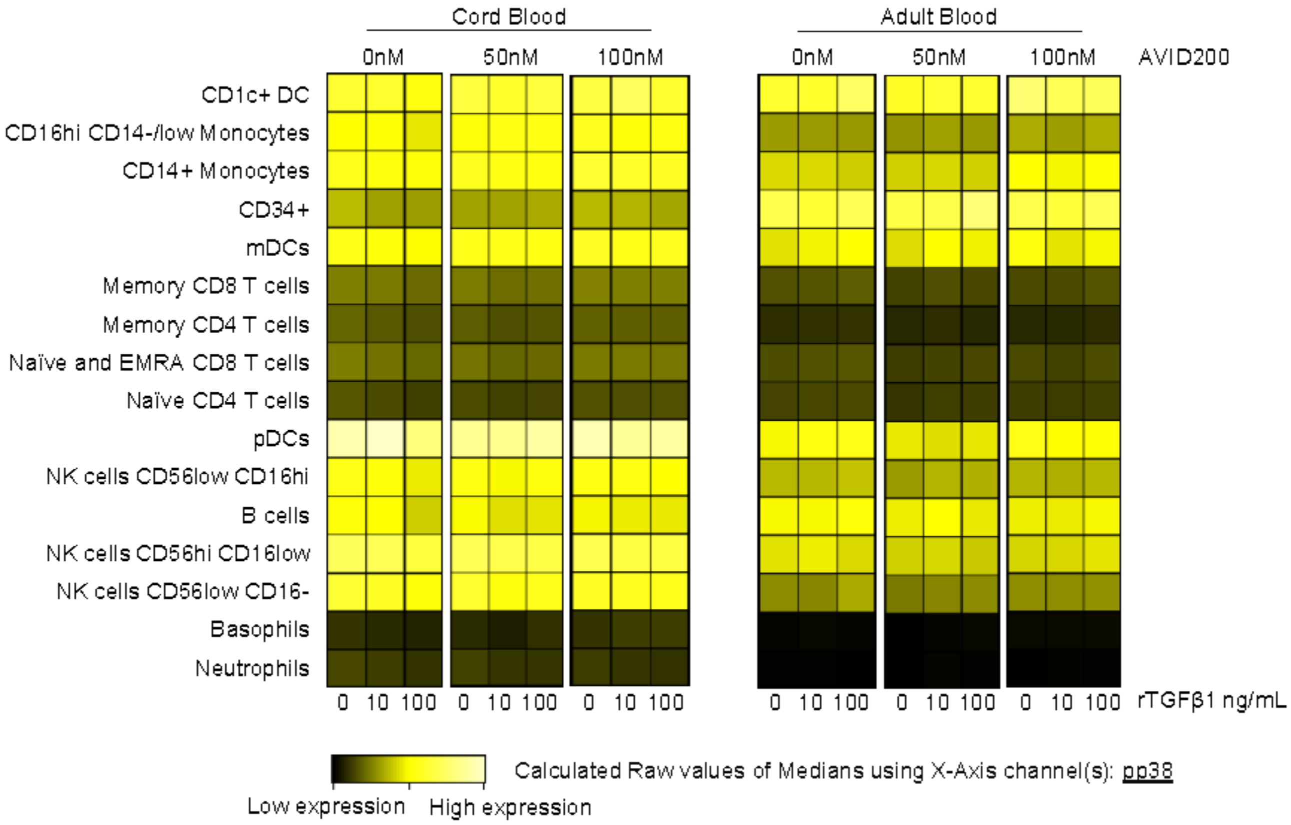

Supplemental Figure 6B. CyTOF analysis reveals that AVID200 does not inhibit the effect of rTGFβ1 on pp38 levels. ViSNE analysis for pp38 expression levels in different gated cell populations from healthy donor whole blood (cord and adult blood) treated with rTGFβ1 and AVID200 alone or in combinations at increasing concentrations.

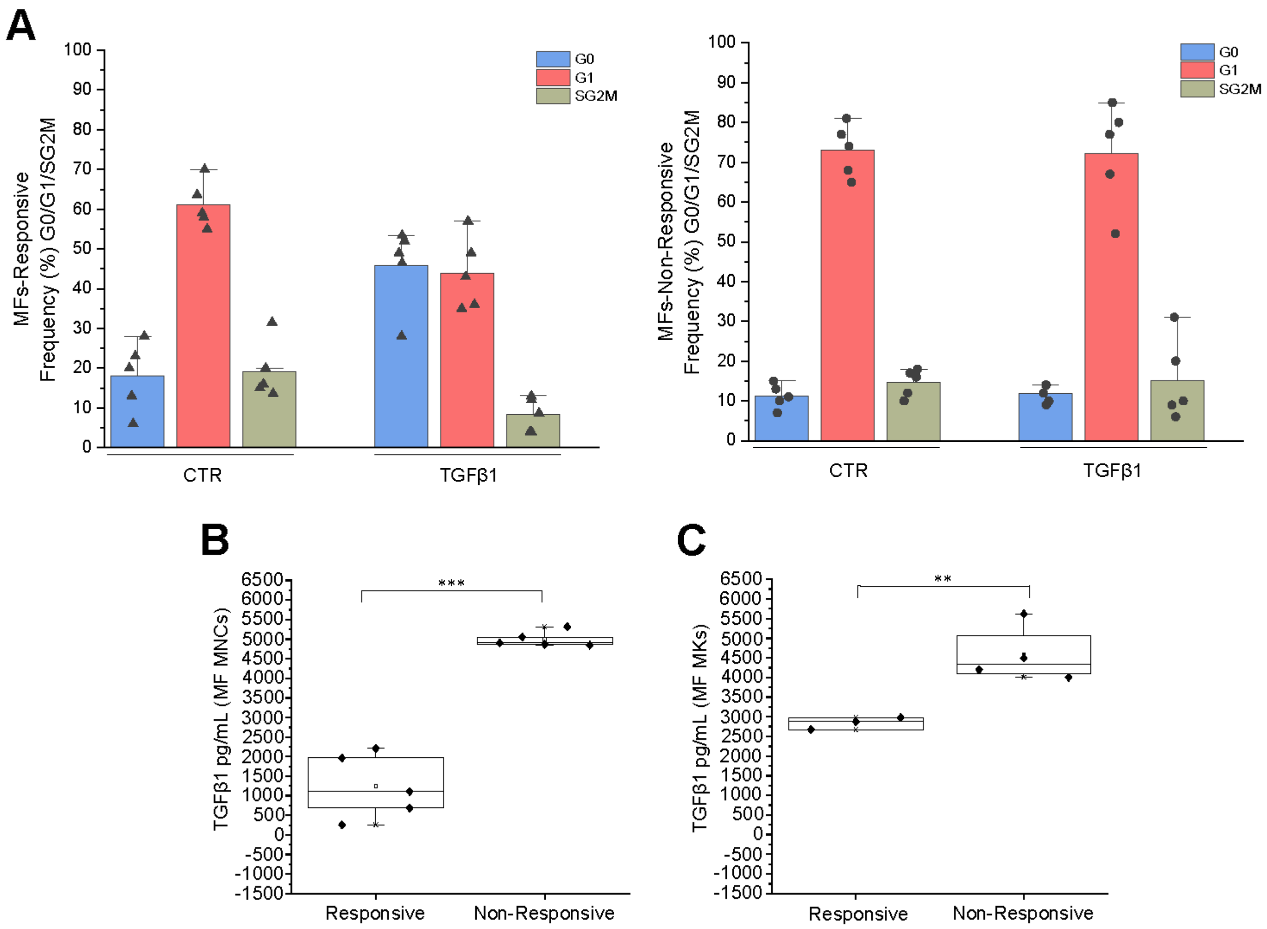

Supplemental Figure 7A-C. Non-responsive MF secrete higher levels of TGFβ1. A) MF responsive (left panel) and non-responsive (right panel) to rTGFβ1 stained with Hoechst 33342 and Pyronin Y. The bar graphs show the percentages ±SD of MF MNCs in G0, G1, and SG2M untreated cells (CTR) or treated with 10ng/mL rTGFβ1 for 48 hours as evaluated by staining with Hoechst 33342 and Pyronin Y (n=5 biological replicates). **B)** The levels of TGFβ1 were quantified in CM by MF cultured as MNCs (day 6) or MK differentiation (day 11). Results are displayed as pg/mL of culture supernatant ±SD (n=5 MNCs; n=4 MKs). Significantly greater levels of TGFβ1 were observed in non-responsive MF HSCs and MKs CM. (***p*< 0.01; ****p* < 0.001 by Anova).

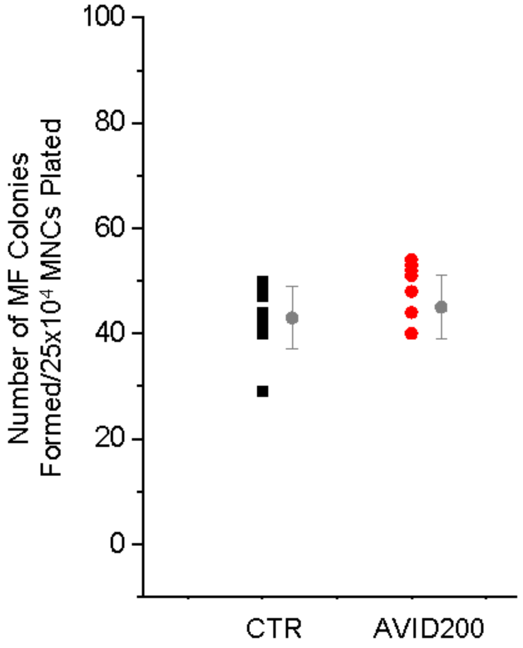

Supplemental Figure 8. AVID200 treatment had no effect on the number of MF hematopoietic colonies assayed. Numbers of hematopoietic colonies assayed from 25×10^4 *JAK2V617F*$^+$ MF-MNCs were cultured for 14 days with a cytokine cocktail in absence (CTR) or presence of 50nM AVID200.

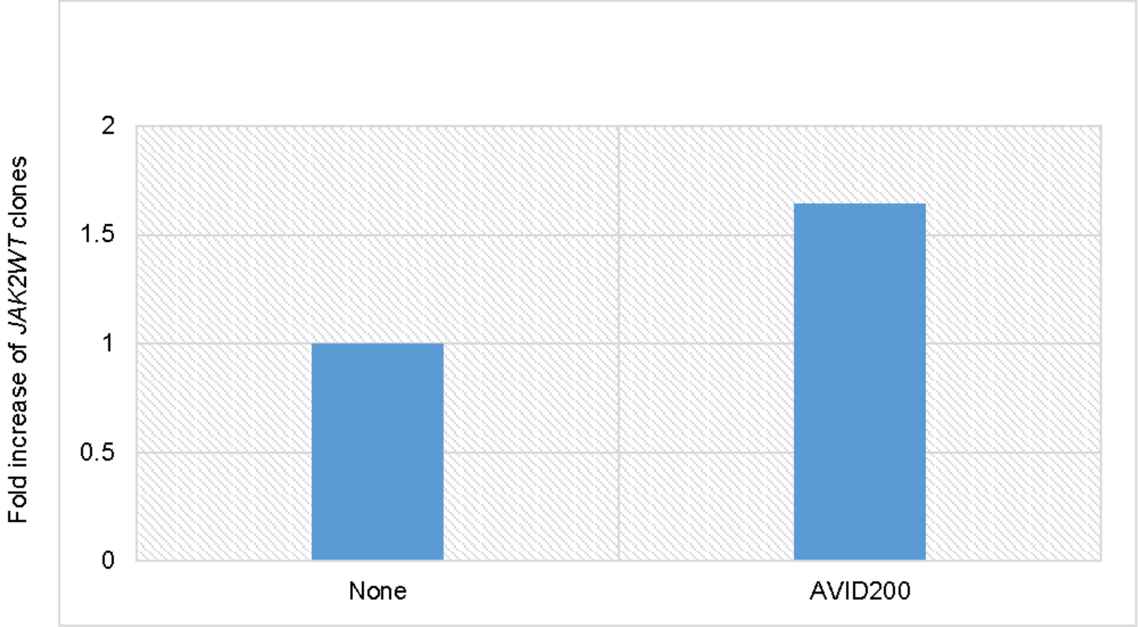

Supplemental Figure 9. AVID200 treatment increases the number of MF *JAK2 wild type* hematopoietic colonies. Fold increase of wild type (WT) colonies assayed from MF-MNCs genotyped for *JAK2V617F* in absence or presence of 50nM AVID200.

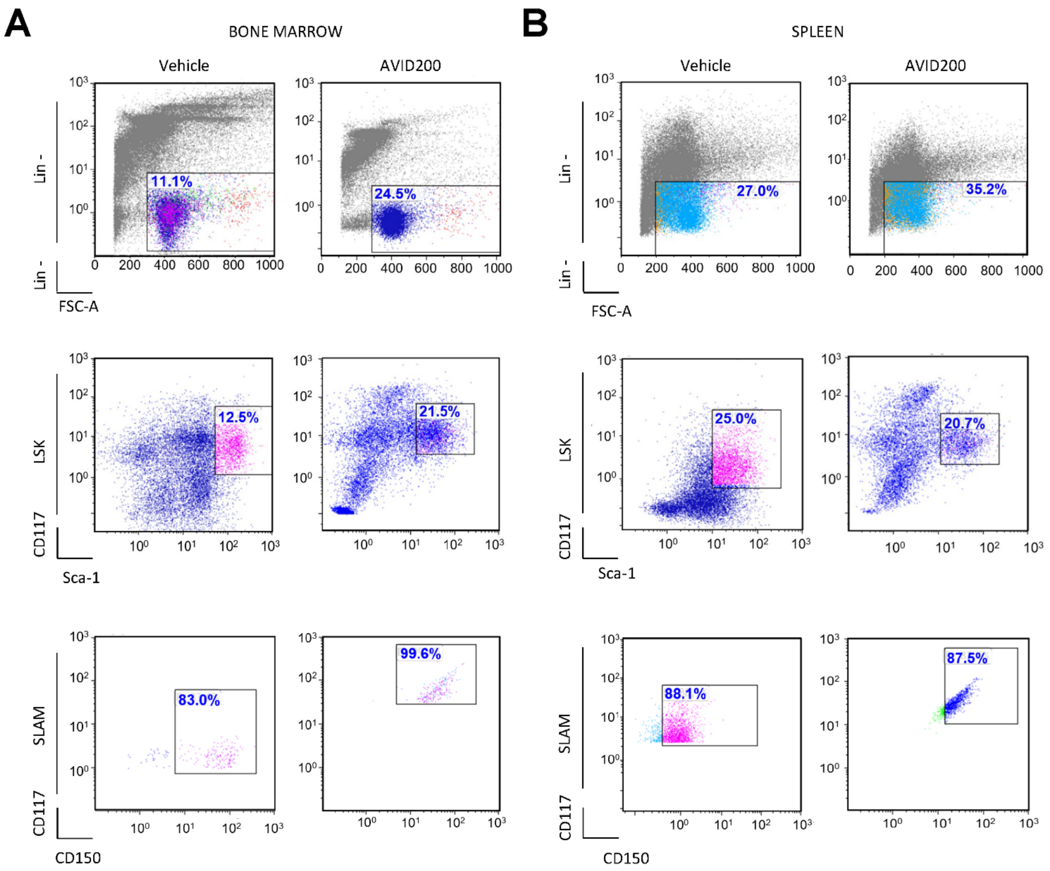

Supplemental Figure 10. Representative sequence of gating used to identify the Lin-, LSK and SLAM (CD150^{pos}/CD48^{neg} LSK) cells in bone marrow **(A)** and spleen **(B)** of *Gata1^{low}* mice treated with either vehicle of AVID200, as indicated.

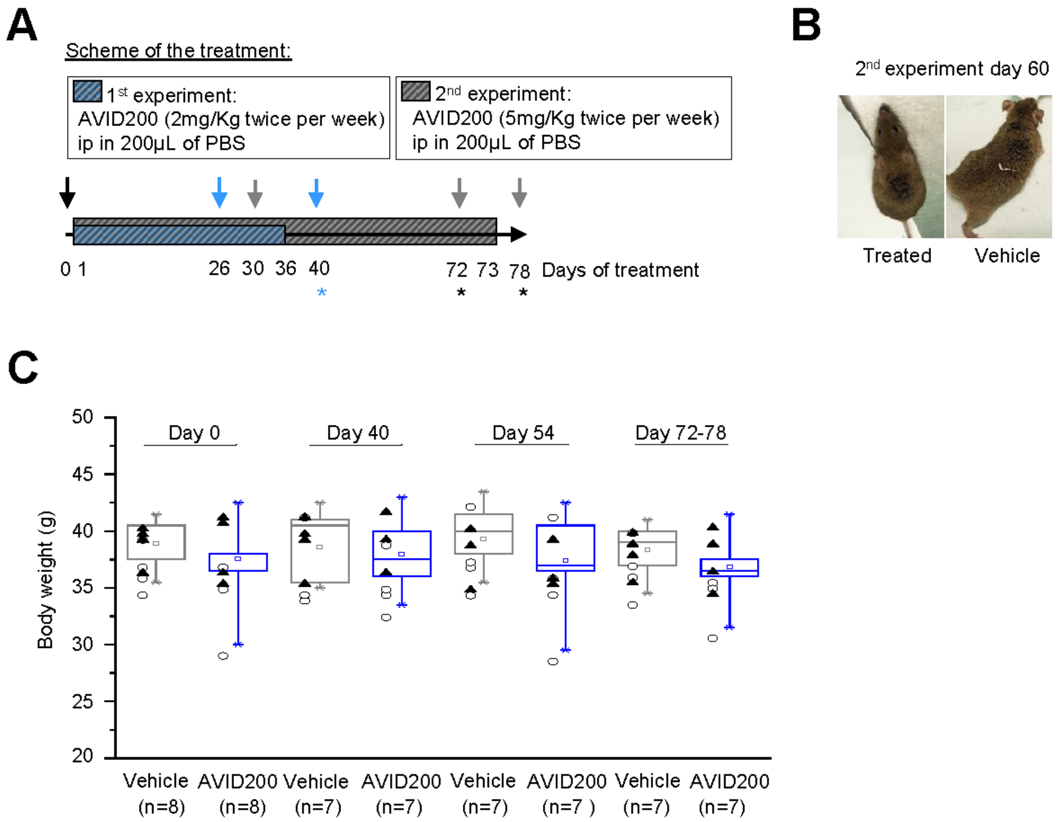

Supplemental Figure 11. Treatment with AVID200 does not affect the body weight of Gata1^{low} mice. A) Scheme of the two treatments. Mice were treated daily either with 2mg (Kg of body weight of AVID200 for 40 days (experiment 1) or with 5mg/Kg of body weight of AVID200 for 73 days. The results of the two experiments are very similar and only those of the second experiment are shown because more complete. Arrows indicate the date of phlebotomy for blood counts and asterisks indicate the date of the sacrifice. **B)** Photographs of representative Gata1low mice at the end of the second experiments. To be noted that the coat of the AVID200 treated mouse is more luster than that of the animal in the vehicle group. **C)** Body weight of mice from the AVID200 and vehicle groups at day 0, 40, 54 and 72-78 of the second experiment.

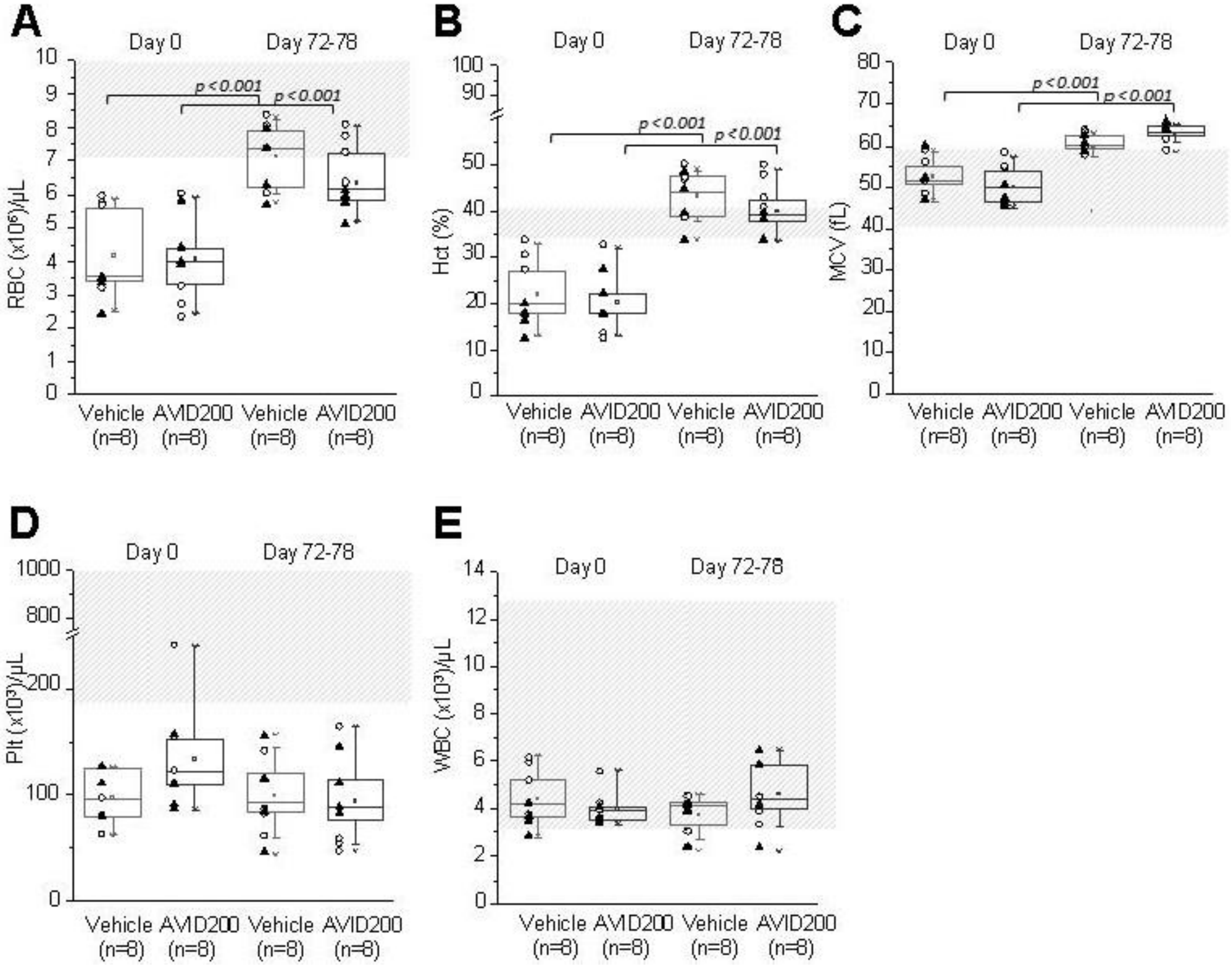

Supplemental Figure 12. Comparison of RBC (A), Hct (B), MCV (C), Ptl (D) and WBC (E) at day 0 and day 72 of Gata1^{low} mice treated either with vehicle or with AVID200, as indicated. The horizontal shaded areas indicate the ranges measured in normal mice by accredited laboratory that performed the measurements.
